# Chloroplasts play a central role in facilitating MAMP‐triggered immunity, pathogen suppression of immunity and crosstalk with abiotic stress

**DOI:** 10.1111/pce.14408

**Published:** 2022-08-05

**Authors:** Susan Breen, Rana Hussain, Emily Breeze, Hannah Brown, Ibrahim Alzwiy, Sara Abdelsayed, Trupti Gaikwad, Murray Grant

**Affiliations:** ^1^ School of Life Sciences University of Warwick Coventry UK; ^2^ School of Biosciences, College of Life and Environmental Sciences University of Exeter Exeter UK; ^3^ Botany Department, Faculty of science Benha University Benha Egypt; ^4^ Present address: Department of Health and Social Care Victoria Street, London SW1H 0EU, UK; ^5^ Present address: Authority of Natural Science Research and Technology P.O. Box 30666, Tripoli, Libya; ^6^ Present address: Marine Biology Association Plymouth PL1 2PB, UK

**Keywords:** chlorophyll fluorescence, chloroplast reactive oxygen, pattern recognition receptors, plant immunity

## Abstract

Microbe‐associated molecular pattern (MAMP)‐triggered immunity (MTI) research has traditionally centred around signal transduction pathways originating from activated membrane‐localized pattern recognition receptors (PRRs), culminating in nuclear transcription and posttranslational modifications. More recently, chloroplasts have emerged as key immune signalling hubs, playing a central role in integrating environmental signals. Notably, MAMP recognition induces chloroplastic reactive oxygen species (cROS) that is suppressed by pathogen effectors, which also modify the balance of chloroplast‐synthesized precursors of the defence hormones, jasmonic acid, salicylic acid (SA) and abscisic acid. This study focuses on how well‐characterized PRRs and coreceptors modulate chloroplast physiology, examining whether diverse signalling pathways converge to similarly modulate chloroplast function. Pretreatment of receptor mutant plants with MAMP and D(Damage)AMP peptides usually protect against effector modulation of chlorophyll fluorescence and prevent *Pseudomonas syringae* effector‐mediated quenching of cROS and suppression of maximum dark‐adapted quantum efﬁciency (the ratio of variable/maximum fluorescence [*F*_v_/*F*_m_]). The MTI coreceptor double mutant, *bak1‐5/bkk1‐1*, exhibits a remarkable decrease in *F*_v_/*F*_m_ compared to control plants during infection, underlining the importance of MTI‐mediated signalling in chloroplast immunity. Further probing the role of the chloroplast in immunity, we unexpectedly found that even moderate changes in light intensity can uncouple plant immune signalling.

## 1 INTRODUCTION

The plant immune system is multilayered and complex. It traditionally comprises three modules: microbe‐associated molecular pattern (MAMP)‐triggered immunity (MTI), effector‐triggered immunity (ETI) and systemic acquired resistance (Jones & Dangl, 2006; Shine et al., 2019). The initial layer of defence, MTI, provides broad‐spectrum defence against a diverse range of pathogens and has recently been shown to be involved in potentiating ETI responses, which can in turn reinforce MTI (Lu & Tsuda, 2021; Ngou et al., 2021; Nguyen et al., 2021; Yuan et al., 2021). Classical pathogen cell surface receptors comprise transmembrane receptor‐like kinases (RLKs) or receptor‐like proteins, including FLAGELLIN SENSING 2 (FLS2), EF‐Tu RECEPTOR (EFR) and CHITIN ELICITOR RECEPTOR KINASE 1 (CERK1‐2), which detect flagellin and elongation factor thermo‐unstable (EF‐Tu) from bacterial pathogens and chitin from fungi, respectively (Yu et al., 2017). However, an increasing number of MAMPs associated with a diverse range of pathogens have been identified (Noman et al., 2019). In addition, cell surface receptors can detect plant‐derived damage‐associated molecular patterns (DAMPs) found within extracellular spaces. Among DAMP receptors are the well‐characterized RLKs, PEP RECEPTOR 1 (PEPR1) and PEPR2, which detect plant elicitor peptides, Peps. PEPR1 recognizes Peps1–6, while PEPR2 recognizes only Pep1 and Pep2 (Yamaguchi et al., 2006, 2010). These Peps are cleaved from the C‐terminus of plant PROPEPs during cell damage and the transcripts of PROPEP1‐3 are induced by defence‐related hormones methyl salicylate and methyl jasmonate (Huffaker et al., 2006; Yamaguchi et al., 2010).

The pattern recognition receptors (PRRs), FLS2, EFR and PEPR1/2, are cell membrane‐localized and contain extracellular leucine‐rich repeat (LRR) surfaces where their ligands bind. Upon peptide detection by PRRs, coreceptors are recruited and bind to PRRs (and in some cases the ligand). The well‐characterized coreceptor brassinosteroid‐insensitive 1 (BRI1)‐associated receptor kinase 1 (BAK1) belongs to the somatic embryogenesis RLK family (SERK), which contains five members, one of which, SERK4/BKK1 (BAK1‐LIKE 1), has high sequence similarity to BAK1 and has functional redundancy (He et al., 2007). While BAK1 was first identified as a coreceptor for the BR receptor BRI1, involved in cell growth and division, it has become widely known for its role in plant immunity as plants containing the reduced function *bak1‐5* allele have impaired FLS2, EFR and PEPR receptor function (Roux et al., 2011; Schwessinger et al., 2011). In contrast, *bkk1‐1* still exhibits a reactive oxygen species (ROS) burst and mitogen‐activated protein kinase (MAPK) (MPK3, MPK4 and MPK6) activation, which is comparable to wild‐type plants, when treated with flg22 or elf18. However, the *bak1‐5/bkk1‐1* plants show minimal ROS and no MAPK activation in response to these MAMPs (Roux et al., 2011; Zipfel et al., 2006).

MTI triggers rapid calcium signalling, ROS and MAPK signalling cascades, all of which involve plasma membrane to nuclear signalling (Noman et al., 2019). Microbes successful in colonization secrete effectors to inter‐ or intracellular locations, which can dampen MTI signalling. Examples of such effector‐triggered suppression (ETS) include the AvrPto effector from *Pseudomonas syringae*, which interacts with the PRRs FLS2 and EFR to dampen MTI in *Arabidopsis thaliana* (Xiang et al., 2008) and AvrE from *P. syringae* and the maize pathogen *Pantoea stewartii* subsp. *Stewartia*, which targets protein phosphatase 2 (PP2A) complexes to dampen MTI (Jin et al., 2016).

Effectors collectively target an array of plant immune signalling components, many of which still remain elusive. Some effectors are directly or indirectly recognized by cytoplasmic receptors, most often belonging to the nucleotide‐binding leucine‐rich repeat receptors (NLRs) class, activating a second immune response, ETI (Jones & Dangl, 2006). There are three major classes of NLRs, the first two classically defined by their N‐terminal: Toll‐like, interleukin‐1 receptor domain TIR‐NLRs (TNLs) and coiled‐coil domain CC‐NLRs (CNLs). More recently, the resistance to powdery mildew 8 CC‐NLR class (Jones et al., 2016; Zhong & Cheng, 2016) have been described, which act as ‘helper’ NLRs for TNL and CNL ‘sensor’ NLRs (Lu & Tsuda, 2021; Maruta et al., 2022; Nguyen et al., 2021). Interaction of an effector and NLR is usually associated with the macroscopic development of the hypersensitive response, which restricts pathogen growth.

Classically, MTI research has centred around signal transduction pathways originating from the plasma membrane and activating nuclear transcription; however, it is becoming increasingly recognized that chloroplasts are a key hub of immune signalling (Kachroo et al., 2021; Littlejohn et al., 2021). Chloroplasts play a central role in integrating environmental signals and maintaining cellular homeostasis via retrograde signalling (Breeze & Mullineaux, 2022; de Souza et al., 2017). Relevant to host immune signalling, chloroplasts are also the site of chloroplastic ROS (cROS) generation and synthesis of defence hormone precursors, jasmonic acid (JA), salicylic acid (SA) and abscisic acid (ABA) (Littlejohn et al., 2021). A key early MTI response is the rapid ROS generation, an apoplastic localized respiratory burst, primarily generated by RBOHD, a member of the NADPH oxidase homolog (RBOH) family (Miller et al., 2009). Activating MTI using an effector secretion deficient strain of *P. syringae* pv. *tomato* strain DC3000 (DC3000*hrpA*) also rapidly generates cROS production in *A. thaliana*, which is attenuated in the virulent DC3000 strain, shortly after effector delivery (de Torres Zabala et al., 2015).

Concomitant with differences in cROS production during infection between the *P. syringae* strains DC3000 and DC3000*hrpA*, global transcriptome profiling of *A. thaliana* revealed significant alterations of nuclear‐encoded chloroplast genes (*NECG*s). Remarkably, *NECGs* represent ~10% of all differentially up‐regulated genes and ~30% of those significantly down‐regulated (de Torres Zabala et al., 2015) during early MTI responses despite *NECGs* collectively accounting for only ~14% of the transcriptome. Superimposed on this, effector delivery (2–3 h postinfection; hpi) caused transcriptional reprogramming of *NECGs*, suggesting ETS also targets *NECG* expression (de Torres Zabala et al., 2015). These molecular signatures are reflected by physiological changes between DC3000 and DC3000*hrpA* challenge as evidenced by quantifying net photosynthetic CO_2_ assimilation (*A*_sat_) and chlorophyll ﬂuorescence imaging parameters associated with electron transport during photosynthesis. DC3000 but not DC3000*hrpA* challenge induced a decrease in CO_2_ assimilation, maximum dark‐adapted quantum efﬁciency (the ratio of variable/maximum fluorescence [*F*_v_/*F*_m_]), maximum operating efﬁciency of photosystem II (PSII) (*F*_v_′/*F*_m_′) and the efﬁciency with which light absorbed by PSII is used for quinone acceptor (QA) reduction and linear electron transport (*F*_q_′/*F*_m_′) (de Torres Zabala et al., 2015). In addition, DC3000 infection elicited an increase in nonphotochemical quenching (NPQ) and PSII redox state (qL; (*F*_q_′/*F*_v_′*)*/(*F*_o_′/*F*′)) compared to DC3000*hrpA* (de Torres Zabala et al., 2015). qL estimates the percentage of open PSII centres and the oxidation state of the primary PSII QA (Baker, 2008). An increase in qL suggests a decrease in electron transport from PSII. Thus, virulent pathogens can radically alter chloroplast physiological functions as part of their virulence strategy.

De novo induction of the plant hormone ABA during DC3000 infection contributes to ETS (de Torres Zabala et al., 2007) and was also recently shown to play a significant role in modulating chloroplast function. DC3000‐induced suppression of *F*_v_/*F*_m_ was accelerated by coinfiltration of 10 µM ABA, effectively phenocopying DC3000 challenge of the *Arabidopsis* ABA hypersensitive protein phosphatase 2C (PP2C) *abi1/abi2/hab1* triple mutant. By contrast, the ABA‐deﬁcient *Arabidopsis aldehyde oxidase 3* (*aao3*) mutant restricted DC3000 suppression of *F*_v_/*F*_m_ (de Torres Zabala et al., 2015). Collectively, these data show that the chloroplast is targeted early in pathogen infection and before bacterial multiplication, with one of the earliest initial events being suppression of cROS.

This study focussed on how well‐characterized MTI PRRs and coreceptors impacted chloroplast physiology, including accessing whether diverse signalling pathways converged to similarly modulate chloroplast function, using *F*_v_/*F*_m_ as the primary readout. Here, we comprehensively examine chlorophyll fluorescence dynamics and the impact on attenuating chloroplast cROS. We show that pretreatment of receptor mutant plants with MAMP and DAMP peptides generally offer protection against effector modulation of chlorophyll fluorescence, but surprisingly, *fls2* plants pretreated with chitin fail to provide such protection. The double mutant of the MTI coreceptors *bak1‐5/bkk1‐1* exhibits a remarkable decrease in *F*_v_/*F*_m_ compared to control plants during infection, highlighting the importance of MTI‐mediated signalling in underpinning chloroplast immunity. Expanding these findings to better understand the role of ABA and abiotic stress in chloroplast immunity, we unexpectedly found that moderate light, representative of that found in the plant's natural environment outside the laboratory, overrides the protection offered by MAMPs on wild‐type plants.

## 2 MATERIALS AND METHODS

### 2.1 *Arabidopsis* growth conditions

*Arabidopsis thaliana* seeds were sown in a compost mix comprising Levingston F2 compost + sand (LEV206):vermiculite (medium grade) mixed in a 6:1 ratio. Plants were grown in a controlled environment growth chamber under 10‐h day (21°C; 120  µmol m^−2^ s^−1^) and 14‐h night (21°C) with a relative humidity of 65% for 5–6 weeks before use.

### 2.2 *Arabidopsis* peptide treatment

Pretreatment of plants was conducted 16 h before bacterial challenge, by infiltration of the peptide. Coinfiltration experiments were conducted by mixing the peptide or hormone of interest with the bacterial culture to attain the required final concentration and OD_600_ before infiltration. Concentrations of peptides or hormones were as follows: 1 µM of flg22, elf18, Pep1, Pep2 and Pep3; 100 µg ml^−1^ of chitin (Sigma; C9752) and 10 or 100 µM ABA. H_2_O was used as a mock for pretreatment.

### 2.3 Bacterial growth, maintenance and inoculation

*Pseudomonas syringae* strains were grown on solid Kings B media containing appropriate antibiotics as described (King et al., 1954; Truman et al., 2006). For inoculation, overnight cultures were grown with shaking (200 rpm) at 28°C. Cells were harvested (1500*g* × 7 min), washed and resuspended in 10 mM MgCl_2_. Cell density was adjusted to OD_600_ 0.15 (∼0.75 × 10^8^ colony‐forming units ml^−1^) for ﬂuorescence imaging and confocal microscopy or OD_600_ 0.0002 for growth assays. Bacteria were infiltrated into the leaves on the abaxial side using one infiltration site on each side of the midvein. All growth assays and ROS imaging experiments were performed at least three times. All ﬂuorescence imaging experiments were performed at least four times.

### 2.4 Chlorophyll ﬂuorescence imaging

PSII chlorophyll ﬂuorescence imaging of *Arabidopsis* rosettes was performed with a CF Imager (Technologica Ltd.). *Normal light cycle*: Plants were placed in the chamber for 40 min postinoculation and then dark adapted for 20 min. This was followed by a saturating light pulse (6349 µmol m^−2^ s^−1^ for 0.8 s) to obtain maximum dark‐adapted ﬂuorescence (*F*_m_). Actinic light (120 µmol m^−2^ s^−1^—the same as plant growth light intensity) was then applied for 15 min, followed by a saturating pulse to obtain maximum light adapted ﬂuorescence (*F*_*m*_′). The plants remained in actinic light for a further 24 min and then returned to a dark period of 20 min. This cycle (59 min duration) was repeated 23 times. *Moderate light cycle*: plants were placed in the chamber for 40 min postinoculation and then dark adapted for 20 min. This was followed by a saturating light pulse (6349 µmol m^−2^ s^−1^ for 0.8 s) to obtain maximum dark‐adapted ﬂuorescence (*F*_m_). Moderate light (650 µmol m^−2^ s^−1^) was then applied for 15 min, followed by three saturating light pulses 5 min apart to obtain maximum light‐adapted ﬂuorescence (*F*_*m*_′). The plants remained in moderate light for a further 150 min and then were returned to a 20 min dark phase. This cycle (200 min duration) was repeated eight times. *F*_m_, *F*_m_′ and *F*_o_ (minimal ﬂuorescence with fully oxidized PSII centres) were used to calculate chlorophyll ﬂuorescence parameters related to PSII: *F*_v_/*F*_m_ (maximum dark‐adapted quantum efﬁciency) and NPQ. These values were calculated as described by (Baker, 2008).

### 2.5 Bacterial growth measurements

Three leaves per plant (six plants total) were syringe infiltrated on the abaxial side using one infiltration site on each side of the midvein with bacteria, OD_600_ 0.0002 and placed either under moderate light (450 or 600 µmol m^−2^ s^−1^) or normal light (120 µmol m^−2^ s^−1^) for 4 days. Three independent leaf discs per plant were excised and homogenized using a Tissue Lyser (Qiagen). Serial dilutions were spotted on Kings B media and colonies were counted at 24 hpi.

### 2.6 Confocal microscopy

Col‐0 plants were pretreated with either water or peptide 16 h before bacterial challenge and then 3.5 hpi leaves were detached and ﬂoated, adaxial surface upwards, in a solution of 10 mM MgCl_2_ containing 10 μM (Enzo) for 40min and then washed for 20 min in 10 mM MgCl_2_ before imaging. Samples were mounted in perﬂuorodecalin (Littlejohn et al., 2010) and images were captured on a Zeiss 880 using a ×40 oil immersion lens. Argon laser excitation at 488 nm and an emission window of 512–527 nm was used to capture the dichloroﬂuorescein (DCF) signal. Chloroplast ﬂuorescence was measured at 659–679 nm.

## 3 RESULTS

### 3.1 MAMP pretreatment protects *F*_v_/*F*_m_ suppression by *P. syringae* DC3000 infection

**Figure 1:**
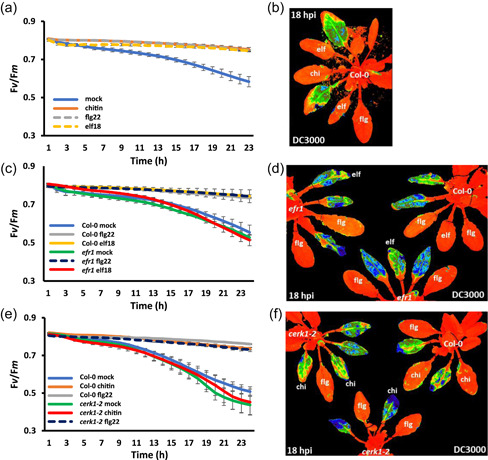
Microbe‐associated molecular pattern (MAMP) pretreatment protects the ratio of variable/maximum fluorescence (*F*_v_/*F*_m_) from bacterially induced suppression. MAMP pretreatment was infiltrated 16 h before bacterial challenge with bacteria infiltrated into the leaves on the abaxial side using one infiltration site on either side of the leaf midvein. (a) Graph quantifying changes in *F*_v_/*F*_m_ over 23 hours postinfection (hpi) with DC3000 infection on Col‐0 leaves. Blue line represents leaves pretreated with H_2_O; orange—pretreated with chitin (100 µg ml^−1^); dashed grey—leaves pretreated with flg22 (1 µM) and dashed yellow—leaves pretreated with elf18 (1 µM). (b) The 18 hpi false‐coloured image *F*_v_/*F*_m_ of a Col‐0 plant pretreated with H_2_O, chitin (100 µg/ml), flg22 (1 µM) and elf18 (1µM) at 18 hpi with DC3000. Orange represents normal *F*_v_/*F*_m_, whereas yellow/green/blue represents suppressed *F*_v_/*F*_m_. (c) Graph quantifying changes in *F*_v_/*F*_m_ over 24 h following DC3000 infection on Col‐0 and *efr1* leaves. Blue line represents Col‐0 leaves pretreated with H_2_O; grey—Col‐0 leaves pretreated with flg22 (1 µM); yellow—Col‐0 leaves pretreated with elf18 (1 µM); green—*efr1* leaves pretreated with H_2_O; dashed blue—*efr1* leaves pretreated with flg22 (1 µM); red—*efr1* leaves pretreated with elf18 (1 µM). (d) False‐coloured visual snapshot of *F*_v_/*F*_m_ for a Col‐0 plant (top right) and *efr1* plants pretreated with H_2_O, flg22 (1 µM) and elf18 (1 µM) at 18 hpi with DC3000. Orange represents normal *F*_v_/*F*_m_, whereas yellow/green/blue represents suppressed *F*_v_/*F*_m_. (e) Graph quantifying changes in *F*_v_/*F*_m_ over 24 h of DC3000 infection on Col‐0 and *cerk1‐2* leaves. Blue represent Col‐0 leaves pretreated with H_2_O; orange—Col‐0 leaves pretreated with chitin (100 µg ml^−1^); grey—Col‐0 leaves pretreated with flg22 (1 µM); green—*cerk1‐2* leaves pretreated with H_2_O; red—*cerk1‐2* leaves pretreated with chitin (100 µg ml^−1^) and dashed blue—*cerk1‐2* leaves pretreated with flg22 (1 µM). (f) The 18 hpi false‐coloured image of *F*_v_/*F*_m_ for Col‐0 (top right) and *cerk1‐2* plants pretreated with H_2_O, flg22 (1 µM) and chitin (100 µg ml^−1^) challenged with DC3000. Orange represents normal *F*_v_/*F*_m_, whereas yellow/green/blue represents suppressed *F*_v_/*F*_m_. Flg22; flg: chitin; chi: elf18; elf. [Color figure can be viewed at wileyonlinelibrary.com]

Previous work showed that leaves pretreated with flg22 24 hpi with virulent *P. syringae* DC3000‐restricted effector‐induced suppression of maximum dark‐adapted quantum efﬁciency (*F*_v_/*F*_m_) levels (de Torres Zabala et al., 2015). To determine if this observation was true for other peptide elicitors, wild‐type, Col‐0, *A. thaliana* leaves were pretreated with the bacterial peptides flg22 (1 µM), elf18 (1 µM) and the fungal peptide chitin (chi; 100 µg ml^−1^) and then challenged with DC3000 at 16 hpi. All pretreatment‐protected challenged leaves from DC3000‐induced suppression of *F*_v_/*F*_m_ over a 24‐h period (Figure 1a). Figure 1b illustrates *F*_v_/*F*_m_ images at 18 hpi showing pretreated leaves (flg, elf, chi) have healthy *F*_v_/*F*_m_ responses (red/orange false‐coloured leaves), whereas reduced *F*_v_/*F*_m_ due to DC3000 infection following H_2_O (mock) pretreatment is indicated by their distinctive green/blue false colouration.

**Figure 2:**
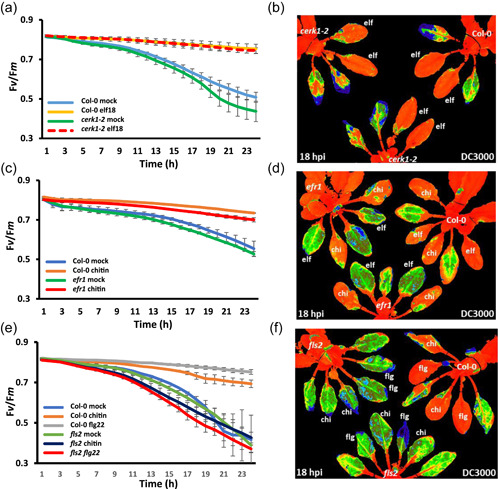
Microbe‐associated molecular pattern (MAMP) pretreatment protects the ratio of variable/maximum fluorescence (*F*_v_/*F*_m_) from bacterially induced suppression, with the exception of chitin pretreatment of *fls2* leaves. MAMP pretreatment was infiltrated 16 h before bacterial challenge, bacteria were infiltrated into the leaves on the abaxial side using one infiltration site on either side of the leaf midvein. (a) Graph quantifying changes in *F*_v_/*F*_m_ over 24 hours postinfection (hpi) with DC3000 on Col‐0 and *cerk1‐2* leaves. Blue line represents Col‐0 leaves pretreated with H_2_O; yellow—Col‐0 leaves pretreated with elf18 (1 µM); green—*cerk1‐2* leaves pretreated with H_2_O and dashed red line—*cerk1‐2* leaves pretreated with elf18 (1 µM). (b) Representative visual false‐coloured snapshot of *F*_v_/*F*_m_ for a Col‐0 plant (top right) and *cerk1‐2* plants pretreated with H_2_O and elf18 (1 µM) at 18 hpi with DC3000. Orange represents normal *F*_v_/*F*_m_, whereas yellow/green/blue represents suppressed *F*_v_/*F*_m_. (c) Graph quantifying changes in *F*_v_/*F*_m_ over 24 h of DC3000 infection on Col‐0 and *efr1* leaves. Blue represents Col‐0 leaves pretreated with H_2_O; orange—Col‐0 leaves pretreated with chitin (100 µg ml^−1^); green—*efr1* leaves pretreated with H_2_O and red—*efr1* leaves pretreated with chitin (100 µg ml^−1^). (d) Visual false‐coloured snapshot of *F*_v_/*F*_m_ for a Col‐0 plant (top right) and *efr1* plants pretreated with H_2_O and chitin (100 µg ml^−1^) at 18 hpi with DC3000. Orange represents normal *F*_v_/*F*_m_, whereas yellow/green/blue represents suppressed *F*_v_/*F*_m_. (e) Graph quantifying changes in *F*_v_/*F*_m_ over 24 h of DC3000 infection on Col‐0 and *fls2* leaves. Blue represent Col‐0 leaves pretreated with H_2_O; orange—Col‐0 leaves pretreated with chitin (100 µg ml^−1^); grey—Col‐0 leaves pretreated with flg22 (1 µM); green—*fls2* leaves pretreated with H_2_O; dark blue—*fls2* leaves pretreated with chitin (100 µg ml^−1^) and red—*fls2* leaves pretreated with flg22 (1 µM). (f) False‐coloured image of *F*_v_/*F*_m_ for a Col‐0 plant (top right) and *fls2* plants pretreated with H_2_O, chitin (100 µg ml^−1^) and flg22 (1 µM) at 18 hpi with DC3000. Orange represents normal *F*_v_/*F*_m_, whereas yellow/green/blue represents suppressed *F*_v_/*F*_m_. [Color figure can be viewed at wileyonlinelibrary.com]

Flg22, elf18 and chitin are recognized by the plant cell surface PRRs FLS2, EFR and Cerk1‐2, respectively. Elf18 pretreatment of *fls2* leaves primed the plant and this crossprotection resulted in no change to *F*_v_/*F*_m_ during DC3000 infection (de Torres Zabala et al., 2015). These data indicate that activation of different MTI receptors can abrogate effector‐mediated *F*_v_/*F*_m_ suppression. Consistent with this hypothesis, flg22 pretreatment on *efr1* (Figure 1c,d) or *cerk1‐2* leaves (Figure 1e,f) results in a protection against DC3000 mediated *F*_v_/*F*_m_ suppression over a 24 h period. The level of protection offered by flg22 to *efr1* and *cerk1‐2* mutants is comparable to the Col‐0 control (Figure 1c,e). By contrast, pretreatment of elf18 on *efr1* plants (Figure 1c,d) and chitin on *cerk1‐2* plants (Figure 1e,f) failed to prevent suppression of *F*_v_/*F*_*m*_ following DC3000 infection. The *F*_v_/*F*_m_ images at 18 hpi illustrate healthy (red/orange) flg22‐pretreated leaves on Col‐0, *efr1* and *cerk1‐2* plants compared to suppression of *F*_v_/*F*_m_ (green/blue) induced by DC3000 infection (Figure 1d,f) following H_2_O treatment. In addition, elf18 pretreatment protects *cerk1‐2* plants from reduced *F*_v_/*F*_m_ during DC3000 infection (Figure 2a,b). Notably, *cerk1‐2*‐challenged leaves showed greater suppression of *F*_v_/*F*_m_ compared to Col‐0 (Figures 1d and 2a), indicating uncoupling chitin signalling may also weaken chloroplast immune responses. Surprisingly, while pretreatment of *efr1* plants with chitin protected them from DC3000‐induced suppression of *F*_v_/*F*_m_ levels, chitin pretreatment failed to protect *fls2* plants during DC3000 infection (Figure 2c,d), where DC3000 suppression of *F*_v_/*F*_m_ was indistinguishable from H_2_O pretreatment (Figure 2e,f).

### 3.2 MAMP pretreatment compromises effector‐induced suppression of cROS

**Figure 3:**
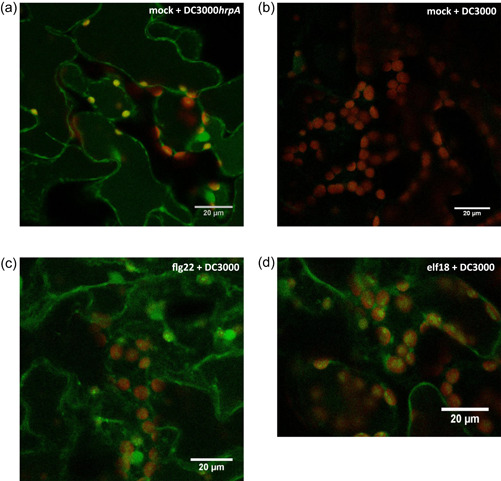
DC3000 is unable to suppress chloroplastic reactive oxygen species in microbe‐associated molecular pattern (MAMP) pretreatment leaves. MAMP pretreatment was infiltrated 16 h before bacterial challenge and bacteria were infiltrated into the leaves on the abaxial side using one infiltration site on either side of the leaf midvein. Col‐0 leaves treated with the nonspecific species stain 2′,7′‐dichlorodihydroﬂuorescein diacetate 5.5 hours postinfection (hpi) with DC3000*hrpA* and DC3000. Leaves were imaged on a Zeiss 880 confocal microscope using excitation at 488 nm and an emission window of 512–527nm to capture the oxidized dichlorofluorescein signal (green). Chloroplast ﬂuorescence was measured at 659–679 nm (red). Scale bars = 20 µm. (a) H_2_O pretreated and DC3000*hrpA* infection. (b) H_2_O pretreated and DC3000 infection. (c) flg22 (1 µM) pretreated and DC3000 infection. (d) elf18 (1 µM) pretreated and DC3000 infection. Image representative of three biological replicates. [Color figure can be viewed at wileyonlinelibrary.com]

cROS are products of photosynthetic electron transport, comprising singlet oxygen (^1^O_2_), hydrogen peroxide (H_2_O_2_) and superoxide anions (O_2_^•−^), with O_2_^•−^ and ^1^O_2_ being produced under high light stress at PSI and PSII respectively (Foyer & Hanke, 2022). The MTI‐induced cROS burst has emerged as an important component of plant immunity, as evidenced by early DC3000 effector delivery to attenuate this process (de Torres Zabala et al., 2015). Therefore, we first assessed the relationship between *F*_v_/*F*_m_ and cROS production and the role of DC3000 effectors in these processes by treating leaves with the nonspecific ROS reporter, 2′,7′‐dichlorodihydroﬂuorescein diacetate (H_2_DCF‐DA) and imaging cROS following DC3000 infection. Strong cROS induction following DC3000*hrpA* infection (MTI) was evident at 4.5 hpi, whereas cROS was minimal in DC3000‐challenged (ETS) leaves at this time (Figure 3a,b). Notably, flg22 or elf18 pretreatment of leaves before DC3000 challenge generated cROS at 4.5 hpi (Figure 3c,d), indicating that the effectors secreted during DC3000 infection could not dampen cROS within a primed leaf.

### 3.3 *bkk1‐1/bak1‐5* plants show hyper suppression of *F*_v_/*F*_m_ during *P. syringae* DC3000 infection

**Figure 4:**
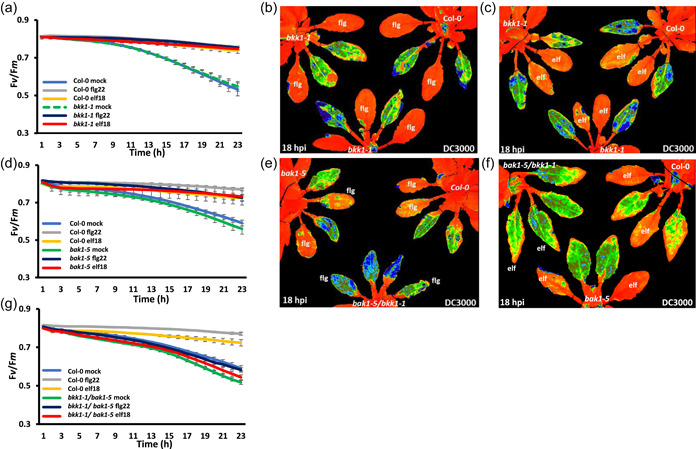
Bacterial microbe‐associated molecular pattern (MAMP) pretreatments provide full and partial protection on *bkk1‐1* and *bak1‐5* single mutant lines but fail to protect *bak1‐5/bkk1‐1* lines. MAMP pretreatment was infiltrated 16 h before bacterial challenge and bacteria were infiltrated into the leaves on the abaxial side using one infiltration site on either side of the leaf midvein. (a) Graph quantifying changes in the ratio of variable/maximum fluorescence (*F*_v_/*F*_m_) over 23 h of DC3000 infection on Col‐0 and *bkk1‐1* leaves. Blue line represents Col‐0 leaves pretreated with H_2_O; grey—Col‐0 leaves pretreated with flg22 (1 µM); yellow—Col‐0 leaves pretreated with elf18 (1 µM); green dashed line—*bkk1‐1* leaves pretreated with H_2_O; dark blue—*bkk1‐1* leaves pretreated with flg22 (1 µM); red—*bkk1‐1* leaves pretreated with elf18 (1 µM). (b) False‐coloured image, 18 hours postinfection (hpi) with DC3000, of *F*_v_/*F*_m_ for Col‐0 (top right) and *bkk1‐1* plants pretreated with H_2_O and flg22 (1 µM). (c) False‐coloured image, 18 hpi with DC3000, of *F*_v_/*F*_m_ for Col‐0 (top right) and *bkk1‐1* plants pretreated with H_2_O and elf18 (1 µM). Orange represents normal *F*_v_/*F*_m_, whereas yellow/green/blue represents suppressed *F*_v_/*F*_m_. (d) Graph quantifying changes in *F*_v_/*F*_m_ over 23 hpi with DC3000 on Col‐0 and *bak1‐5* leaves. Blue represents Col‐0 leaves pretreated with H_2_O; grey—Col‐0 leaves pretreated with flg22 (1 µM); yellow—Col‐0 leaves pretreated with elf18 (1 µM); green—*bak1‐5* leaves pretreated with H_2_O; dark blue—*bak1‐5* leaves pretreated with flg22 (1 µM); red corresponds to *bak1‐5* leaves pretreated with elf18 (1 µM). (e) False‐coloured image at 18 hpi with DC3000, of *F*_v_/*F*_m_ for Col‐0 (top right), *bak1‐5* (top left) or *bak1‐5/bkk1‐1* (bottom), plants pretreated with H_2_O and flg22 (1 µM). (f) False‐coloured image, 18 hpi with DC3000, of *F*_v_/*F*_m_ for Col‐0 (top right), *bak1‐5* (bottom) or *bak1‐5/bkk1‐1* (top left), plants pretreated with H_2_O and elf18 (1 µM) at 18 hpi with DC3000. Orange represents normal *F*_v_/*F*_m_, whereas yellow/green/blue represents suppressed *F*_v_/*F*_m_. (g) Quantitative changes in *F*_v_/*F*_m_ over 23 hpi with DC3000 on Col‐0 and *bak1‐5/bkk1‐1* leaves. Blue represents Col‐0 leaves pretreated with H_2_O; grey—Col‐0 leaves pretreated with flg22 (1 µM); yellow—Col‐0 leaves pretreated with elf18 (1 µM); green—*bak1‐5/bkk1‐1* leaves pretreated with H_2_O; dark blue—*bak1‐5/bkk1‐1* leaves pretreated with flg22 (1 µM); red—*bak1‐5/bkk1‐1* leaves pretreated with elf18 (1 µM). [Color figure can be viewed at wileyonlinelibrary.com]

As primary PRRs mediate chloroplast immune signals, the cell surface coreceptor mutants *bak1‐5*, *bkk1‐1* and double mutant *bkk1‐1/bak1‐5* were used to assess their contribution to altered *F*_v_/*F*_m_ dynamics during DC3000 infection. *bkk1‐1* plants pretreated with flg22, elf18 and chitin 16 h before infection with DC3000 showed *F*_v_/*F*_m_ infection signatures equivalent to those measured following DC3000*hrpA* infection in Col‐0 plants (Figure 4a–c and Supporting Information: Figure 1a,b). DC3000‐challenged *bak1‐5* plants showed a small but significantly greater suppression of *F*_v_/*F*_m_ compared to Col‐0 (Figure 4d), as expected given its partial loss of MTI function (Roux et al., 2011). These data highlight both the power of quantitative chlorophyll fluorescence measurements and the ability to dynamically monitor effector impact on chloroplast physiology. Interestingly, *F*_v_/*F*_m_ dynamics in DC3000*hrpA* challenged *bak1‐5* leaves pretreated with flg22 or elf18 before infection were wild type in response, whereas chitin pretreatment only partially protected against *F*_v_/*F*_m_ suppression in the *bak1‐5* background (Figure 4d–f and Supporting Information: Figure 1c,d). Strikingly, MAMP pretreatment with flg22, elf18 or chitin had no protective effect on *F*_v_/*F*_m_ dynamics in the *bkk1‐1/bak1‐5* double mutant with *F*_v_/*F*_m_ suppression being identical and often greater than the respective Col‐0 control treatment (Figure 4e–g and Supporting Information: Figure 1d,e).

### 3.4 Pretreatment of leaves with DAMPs results in the protection of *F*_v_/*F*_m_ during *P. syringae* DC3000 infection

**Figure 5:**
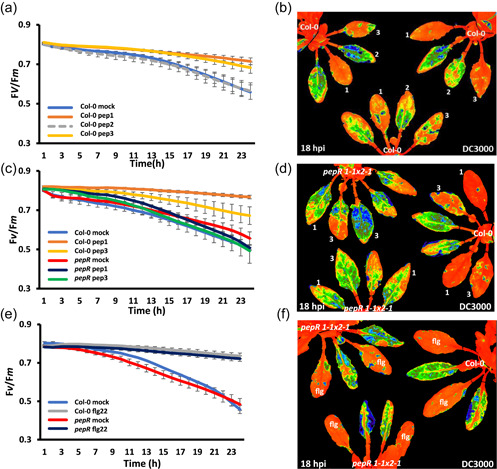
Damage‐associated molecular pattern (DAMP) pretreatment restricts suppression of the ratio of variable/maximum fluorescence (*F*_v_/*F*_m_) following bacterial challenge. DAMP pretreatment was infiltrated 16 h before bacterial challenge and bacteria were infiltrated into the leaves on the abaxial side using one infiltration site either side of the leaf midvein. (a) Graph quantifying changes in *F*_v_/*F*_m_ over 24 hours postinfection (hpi) following DC3000 infection of Col‐0. Blue represents leaves pretreated with H_2_O; orange—pretreated with Pep1 (1 µM); dashed grey—pretreated with Pep2 (1 µM); yellow—pretreated with Pep3 (1 µM). (b) False‐coloured image of *F*_v_/*F*_m_ in Col‐0 plants pretreated with H_2_O, Pep1 (1 µM), Pep2 (1 µM) or Pep3 (1 µM) at 18 hpi with DC3000. Orange represents normal *F*_v_/*F*_m_, whereas yellow/green/blue represents suppressed *F*_v_/*F*_m_. (c) Graph quantifying changes in *F*_v_/*F*_m_ over 24 hpi with DC3000 on Col‐0 and *pepR1‐1 × 1‐2* leaves. Blue represents Col‐0 leaves pretreated with H_2_O; orange—Col‐0 pretreated with pep1 (1 µM); yellow—Col‐0 pretreated with pep3 (1 µM); red—*pepR1‐1 × 1‐2* pretreated with H_2_O; dark blue—*pepR1‐1 × 1‐2* pretreated with pep1 (1 µM); green—*pepR1‐1 × 1‐2* leaves pretreated with pep3 (1 µM). (b) False‐coloured image of *F*_v_/*F*_m_ for Col‐0 (right) and *pepR1‐1 × 1‐2* pretreated with H_2_O, Pep1 (1 µM) and Pep3 (1µM) 18 hpi with DC3000. Orange represents normal *F*_v_/*F*_m_, whereas yellow/green/blue represents suppressed *F*_v_/*F*_m_. (e) Graph quantifying changes in *F*_v_/*F*_*m*_ over 24 hpi with DC3000 on Col‐0 and *pepR1‐1 × 1‐2* leaves. Blue represents Col‐0 leaves pretreated with H_2_O; grey—Col‐0 leaves pretreated with flg22 (1 µM); red—*pepR1‐1 × 1‐2* leaves pretreated with H_2_O and dark blue—*pepR1‐1 × 1‐2* leaves pretreated with flg22 (1 µM). (f) Representative false‐coloured image, 18 hpi with DC3000, of *F*_v_/*F*_m_ for Col‐0 (right) and *pepR1‐1 × 1‐2* plants pretreated with H_2_O and flg22 (1 µM). Orange represents normal *F*_v_/*F*_m_, whereas yellow/green/blue represents suppressed *F*_v_/*F*_m_. [Color figure can be viewed at wileyonlinelibrary.com]

Given the protection offered by MAMPs to *F*_v_/*F*_m_ levels during DC3000 infection, we next tested whether similar protection was also conferred by plant‐derived DAMPs. Using Pep elicitors, Col‐0 leaves were first pretreated with Pep1, Pep2 or Pep3 (all at 1 µM) 16 h before DC3000 challenge. *F*_v_/*F*_m_ dynamics over 24 h revealed that Pep1 and 3 but not Pep2 protected from DC3000 *F*_v_/*F*_m_ suppression (Figure 5a,b). Critically, Pep1 and 3 pretreatment failed to alter DC3000 *F*_v_/*F*_m_ infection dynamics in the *pepR1‐1 × 2‐1* mutant (Yamaguchi et al., 2010) (Figure 5c,d), whereas flg22 pretreated *pepR1‐1 × 2‐1* leaves protected from *F*_v_/*F*_m_ suppression as described above for Col‐0 flg22 pretreatment (Figure 5e,f). Interestingly, Pep1 and Pep3 pretreatment only provided partial protection against *F*_v_/*F*_m_ suppression in DC3000 infected *fls2* leaves and no protection in *bkk1‐1/bak1‐5* plants (Supporting Information: Figure 2a–d). These data indicate a degree of crossprotection of *F*_v_/*F*_m_ between DAMPs and MAMP priming of the plant.

### 3.5 Moderate light enhances *F*_v_/F_m_ reduction during *P. syringae* DC3000 infection

Numerous studies have looked at plant acclimation to high light, typically 1000–3000 μmol m^−2^ s^−1^. Excess light can be absorbed by the light‐harvesting complexes and dissipated as heat via thermal energy dissipation, linked to NPQ mechanisms (Holt et al., 2004). Excess light also modulates both ROS and phytohormones. Crosstalk between ROS‐ and SA‐dependent pathways has been shown to regulate both light acclimation and defence responses leading to pathogen resistance, as reviewed in Kangasjarvi et al. (2012, 2013).

Light levels typically used in *Arabidopsis–*pathogen interactions are significantly lower than those used for studying acclimation to excess excitation energy, tending to be of relatively low light intensity (80–150 mol m^−2^ s^−1^). Outside the laboratory, light levels are usually substantially higher than those and often fluctuating, thus requiring a dynamic response from the photosynthetic apparatus via an array of homeostatic control mechanisms that modulate changes in cellular energy and reductant status (Kangasjarvi et al., 2012) and retrograde signalling (Szechyńska‐Hebda & Karpiński, 2013).

**Figure 6:**
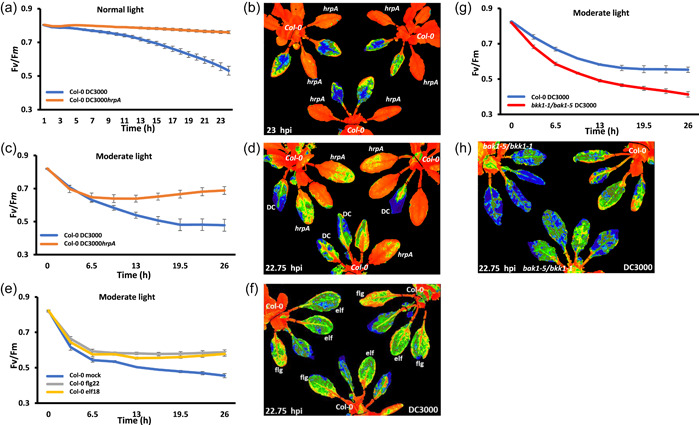
Moderate light enhances bacterial suppression of the ratio of variable/maximum fluorescence (*F*_v_/*F*_m_). Bacteria were infiltrated into the leaves on the abaxial side using one infiltration site on either side of the leaf midvein, where undertaken microbe‐associated molecular pattern (MAMP) Pretreatment was infiltrated 16 h before bacterial challenge. (a) Graph quantifying changes in *F*_v_/*F*_m_ over 24 hours postinfection (hpi) of DC3000 (blue line) and DC3000*hrpA* (orange line) infection on Col‐0 leaves under normal light (NL) (120 µmol m^−2^ s^−1^). (b) False‐coloured image of *F*_v_/*F*_m_ at 23 hpi of Col‐0 plants with DC3000 and DC3000*hrpA* under NL (120 µmol m^−2^ s^−1^). Orange represents the expected *F*_v_/*F*_*m*_, whereas yellow/green/blue represents suppressed *F*_v_/*F*_m_. (c) Graph quantifying changes in *F*_v_/*F*_m_ over 26.5 hpi of DC3000 (blue line) and DC3000*hrpA* (orange line) infection on Col‐0 leaves under moderate light (650 µmol m^−2^ s^−1^). (d) False‐coloured image of *F*_v_/*F*_m_ at 22.75 hpi of Col‐0 plants with DC3000 and DC3000*hrpA* under 650 µmol m^−2^ s^−1^. Unlabelled leaves are not infiltrated. Orange represents expected *F*_v_/*F*_m_, whereas yellow/green/blue represents suppressed *F*_v_/*F*_m_. (e) Graph quantifying changes in *F*_v_/*F*_m_ over 26.5 hpi of DC3000 on Col‐0 under moderate light (650 µmol m^−2^ s^−1^). Blue represents Col‐0 leaves pretreated with H_2_O; grey—Col‐0 leaves pretreated with flg22 (1 µM); yellow line—Col‐0 leaves pretreated with elf18 (1 µM). (f) False‐coloured image of *F*_v_/*F*_*m*_ for Col‐0 plants pretreated with H_2_O, flg22 (1 µM) and elf18 (1 µM) at 22.75 hpi with DC3000 under 650 µmol m^−2^ s^−1^. Orange represents expected *F*_v_/*F*_m_, whereas yellow/green/blue represents suppressed *F*_v_/*F*_m_. (g) Graph quantifying changes in *F*_v_/*F*_m_ over 26.5 hpi of Col‐0 and *bak1‐5/bkk1‐1* leaves with DC3000 under moderate light (650 µmol m^−2^ s^−1^). Blue represents Col‐0 and red *bak1‐5/bkk1‐1* leaves. (h) Image of *F*_v_/*F*_m_ 22.75 hpi with DC3000 on Col‐0 plant (right) or *bak1‐5/bkk1‐1* plants under 650 µmol m^−2^ s^−1^. Orange represents expected *F*_v_/*F*_m_, whereas yellow/green/blue represents suppressed *F*_v_/*F*_m_. [Color figure can be viewed at wileyonlinelibrary.com]

To explore the impact of ‘moderate’ light levels (300–600 μmol m^−2^ s^−1^), we examined *F*_v_/*F*_m_ dynamics during DC3000 infection under standard growth conditions (normal light) or moderate light conditions. A ‘normal’ light (120 µmol m^−2^ s^−1^) cycle of 1 h (as above) comprised 40 min of light before dark adaption for 24 cycles. To ensure the duration of moderate light exposure would encompass early pathogen infection events, including expression of effector genes and assembly of the Type‐III secretion system (T3SS), we used a regime of 2.5 h moderate light (650 µmol m^−2^ s^−1^) before dark adaption, enabling *F*_v_/*F*_m_ measurements to be captured eight times over a 26 h period. In comparison to DC3000 or DC3000*hrpA* challenge under normal light conditions (Figure 6a,b), moderate light resulted in a dramatic initial decrease of *F*_v_/*F*_m_ within the first 6.5 hpi for both DC3000 and DC3000*hrpA* challenges. *F*_v_/*F*_m_ in DC3000*hrpA*‐treated leaves partially recovered and did not regain levels observed under normal light conditions (Figure 6c,d). By contrast, leaves infected with DC3000 showed strong decreases in *F*_v_/*F*_*m*_ over the entire 26 h. These were consistently significantly lower than that observed in infected leaves under normal light (Figure 6a–d). Interestingly, at 650 µmol m^−2^ s^−1^, flg22 and elf18 pretreatment failed to prevent the majority of the suppression of *F*_v_/*F*_m_ and, in fact, showed infection dynamics very similar to that observed in DC3000 challenged Col‐0 leaves. These data imply this moderately increased light alone was sufficient to drive enhanced *F*_v_/*F*_m_ suppression by DC3000 (Figure 6e,f), and strikingly, further suppress the *F*_v_/*F*_m_ infection dynamics observed in DC3000 challenged *bkk1‐1/bak1‐5* leaves (Figure 6g,h).

**Figure 7:**
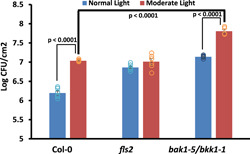
Moderate light renders Col‐0 and *bak1‐5/bkk1‐1* plants more susceptible to bacterial infection. Bacterial growth of DC3000 on Col‐0, *fls2* and *bak1‐5/bkk1‐1* plants under normal light (NL; blue; 120 µmol m^−2^ s^−1^) and moderate light (ML; red; 450 µmol m^−2^ s^−1^). Error bars, mean ± SE (*n* = 6), Student's *t*‐test determined the statistical significance of *p* < 0.0001 for NL Col‐0 versus *fls2* and Col‐0 versus *bak1‐5/bkk1‐1* (not shown on graph), ML Col‐0 versus *bak1‐5/bkk1‐1*, Col‐0 NL versus ML and *bak1‐5/bkk1‐1* NL versus ML. There was no significant difference between ML Col‐0 versus *fls2* and *fls2* NL versus ML. Representative of three biological replicates. [Color figure can be viewed at wileyonlinelibrary.com]

To ascertain the impact of moderate light on host susceptibility, we enumerated bacterial growth under 120 or 450 μmol m^−2^ s^−1^ light regimes. As the strong *F*_v_/*F*_m_ suppression under moderate light exhibited by the DC3000 challenge is reminiscent of ETI responses (Littlejohn et al., 2021), it was surprising that 450 μmol m^−2^ s^−1^ (<4‐fold increase in intensity) enhanced susceptibility (Figure 7 and Supporting Information: Figure 3b). Interestingly, already hypersusceptible *bkk1‐1/bak1‐5* plants were even more susceptible to DC3000 infection at 450 µmol m^−2^ s^−1^, suggesting that this moderate light intensity uncouples immunity through pathways independently of those guarded by classical MTI signalling (Figure 7), and/or that MTI signalling is less effective at increased light intensity. There was, however, no significant difference in bacterial growth observed for *fls2* in comparison to Col‐0 plants under moderate light (Figure 7). This apparent insensitivity of *fls2* plants at moderate light conditions warrants further investigation. Notably, plants preadapted to moderate light were no more or less susceptible than plants exposed to moderate light immediately after the DC3000 challenge (Figure 7 and Supporting Information: Figure 3). Despite visibly showing the presence of anthocyanins, often associated with the accumulation of defensive metabolites (Gould, 2004; Lev‐Yadun & Gould, 2008; Schaefer & Rolshausen, 2006), compared to the cognate control plants under 120 µmol m^−2^ s^−1^ (Supporting Information: Figure 3a), plants that had been acclimatized to moderate light treatment for 5 days showed similar enhanced susceptibility (Supporting Information: Figure 3b). Thus, moderate light preadaptation is not required to elicit enhanced susceptibility, it is only required coincident with pathogen infection to significantly enhance bacterial growth, and this is additional to that achieved by uncoupling classical MTI defences.

### 3.6 Pathogen‐induced suppression of ABA enhances *F*_v_/*F*_m_ reduction

**Figure 8:**
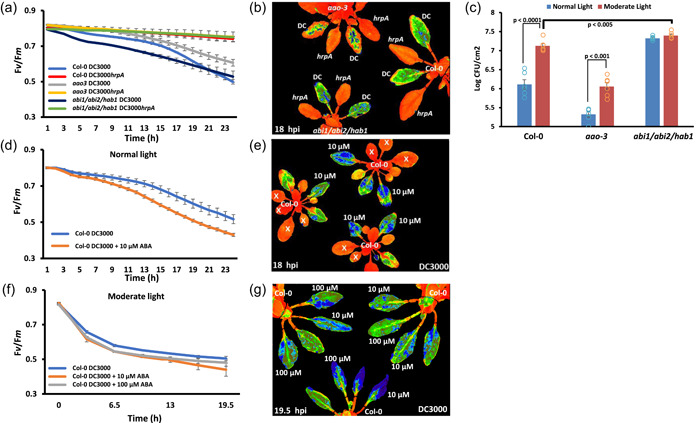
Exogeneous abscisic acid (ABA) synergistically or antagonistically alters pathogen‐induced ABA suppression of the ratio of variable/maximum fluorescence (*F*_v_/*F*_m_) in a concentration‐dependent manner. Bacteria were infiltrated into the leaves on the abaxial side using one infiltration site on either side of the leaf midvein, where undertaken hormones were mixed and coinfiltrated with a bacterial challenge. (a) Graph quantifying changes in *F*_v_/*F*_m_ over 24 hours postinfection (hpi) following DC3000 or DC3000*hrpA* infection of Col‐0, *aao3* and *abi1/abi2/hab1* leaves. Blue represents Col‐0 leaves infiltrated with DC3000; red—Col‐0 leaves infiltrated with DC3000hrpA; grey—aao3 leaves infiltrated with DC3000; yellow—*aao3* leaves infiltrated with DC3000hrpA; dark blue—*abi1/abi2/hab1* leaves infiltrated with DC3000 and green corresponds to abi1/abi2/hab1 leaves infiltrated with DC3000hrpA. (b) False‐coloured image of F_v_/F_m_ of Col‐0, *aao3* and abi1/abi2/hab1 plants 18 hpi with DC3000 or DC3000*hrpA*. Orange represents normal *F*_v_/*F*_m_ readout, whereas yellow/green/blue represents suppressed *F*_v_/*F*_m_. (c) Bacterial growth of DC3000 on Col‐0, *aao3* or *abi1/abi2/hab1* plants under normal light (NL; 120 µmol m^−2^ s^−1^; blue) or moderate light (ML; 450 µmol m^−2^ s^−1^; orange) conditions. Error bars, mean ± SE (*n* = 6) Student's *t*‐test determined statistical significance of *p* < 0.0001 for Col‐0 NL versus ML (shown), statistical significance of *p* < 0.001 for *aao3* NL versus ML and statistical significance of *p* < 0.005 for ML Col‐0 versus *abi1/abi2/hab1*. Representative of three replicated experiments. (d) Graph quantifying changes in *F*_v_/*F*_m_ 24 hpi with DC3000 on Col**‐**0 leaves in the presence of ABA under normal light (NL; 120 µmol m^−2^ s^−1^). Blue—Col‐0 leaves infiltrated with DC3000 and orange—Col‐0 leaves co‐infiltrated with DC3000 + 10 µM ABA. (e) False‐coloured image of *F*_v_/*F*_m_ of Col‐0, plants 18 hpi infiltrated with DC3000 and coinfiltrated with DC3000 and 10 µM ABA under NL. Orange represents expected *F*_v_/*F*_m_, whereas yellow/green/blue represents suppressed *F*_v_/*F*_m_. (f) Graph quantifying changes in *F*_v_/*F*_m_ up to 24 hpi with DC3000 of Col_*‐*_0 leaves in the presence of increasing concentrations of ABA under moderate light (ML; 650 µmolm^−2^ s^−1^). Blue represents Col‐0 leaves infiltrated with DC3000; orange—Col‐0 leaves coinfiltrated with DC3000+ 10 µM ABA and grey corresponds to Col‐0 leaves coinfiltrated with DC3000+ 100 µM ABA. (g) False‐coloured image, 19.5 hpi, of *F*_v_/*F*_m_ of Col‐0 infiltrated with DC3000, coinfiltrated with DC3000 and 10 or 100 µM ABA under 650 µmol m^−2^ s^−1^. Orange represents expected *F*_v_/*F*_m_, whereas yellow/green/blue represents suppressed *F*_v_/*F*_m_. [Color figure can be viewed at wileyonlinelibrary.com]

ABA biosynthesis and signalling are hijacked by DC3000 to suppress immunity (de Torres Zabala et al., 2007, 2009). The impact of ABA mutants on virulence is reflected in *F*_v_/*F*_m_ signatures (de Torres Zabala et al., 2015). As ABA is made predominately in the chloroplasts, we investigated whether moderate light‐induced susceptibility was underpinned by ABA signalling. We monitored the impact of an ABA hypersusceptible signalling mutant (triple mutant) or the ABA‐insensitive biosynthetic mutant *aao3* on infection under normal and moderate light, monitoring both *F*_v_/*F*_m_ and NPQ, the latter measuring the energy released as heat. The *aao3* mutant exhibited less suppression of *F*_v_/*F*_m_ during DC3000 infection compared to Col‐0 plants, reflected also in slightly lower levels of NPQ in comparison to Col‐0 (Figure 8a,b and Supporting Information: Figure 4a,b). By contrast, the hypersensitive triple PP2C mutant (*abi1/abi2/hab1*) shows a faster decrease in *F*_v_/*F*_m_ and a stronger increase in NPQ compared to Col‐0 (Figure 8a,b and Supporting Information: Figure 4a,b). As previously reported (Rubio et al., 2009; de Torres Zabala et al., 2007, 2009) under normal light conditions *aao3* plants are more resistant to DC3000, while the triple PP2C mutant is more susceptible (Figure 8c). Notably, while Col‐0 and *aao3* plants are more susceptible at a light intensity of 450 µmol m^−2^ s^−1^, there was no enhanced susceptibility evident in the triple PP2C mutant (Figure 8c), implying either ABA signalling is important for moderate light‐enhanced susceptibility or the *abi1/abi2/hab1* plants cannot support further bacterial multiplication. In addition, Col‐0 plants grown at 450 µmol m^−2^ s^−1^ show accumulation of ABA after 5 days and also at 9 days postinfection with DC3000 compared to no respective increases in ABA under 120 µmol m^−2^ s^−1^ (Supporting Information: Figure 3c). In contrast, the *aao3* plants do not show an increase in ABA under either light regime (Supporting Information: Figure 3c).

To next assess the interaction of ABA and light on chloroplast function during pathogen infection, 10 µM ABA was coinfiltrated with DC3000 into Col‐0 leaves. Under normal light, 10 µM ABA coinfiltration enhances the decrease in *F*_v_/*F*_m_ levels as previously reported (de Torres Zabala et al., 2015) (Figure 8d,e). Under moderate light, DC3000 coinfiltration with 10 and 100 µM ABA resulted in a faster decrease of *F*_v_/*F*_m_ levels from 3.5 h onwards (Figure 8f,g). To determine whether 10 and 100 µM ABA affected *P. syringae*, DC3000 was plated on Kings B agar containing 0, 10, 50 and 100 µM ABA. Bacterial growth was moderately restricted (*p* < 0.0005) between 10 and 100 µM ABA treatments (Supporting Information: Figure 4c).

## 4 DISCUSSION

We had previously shown that MTI significantly alters the expression of photosynthetic and *NECGs* within the first 2 h of challenge with the T3SS deficient nonpathogenic DC3000*hrpA* (Lewis et al., 2015; de Torres Zabala et al., 2015). Notably, this MTI response results in a strong suppression of *NECGs* yet does not significantly reduce the maximum dark‐adapted quantum efﬁciency (*F*_v_/*F*_m_) of PSII (de Torres Zabala et al., 2015) compared to the mock challenge. In comparison, virulent DC3000 can deliver effectors within 2–3 hpi and strongly suppresses *F*_v_/*F*_m_ as well as attenuating MTI‐induced cROS (de Torres Zabala et al., 2015). Notably, DC3000 significantly reconfigures the expression of *NECG* within 3–4 h of infection (de Torres Zabala et al., 2015), the timing of which coincides with the delivery of effectors into the plant cell.

**Figure 9:**
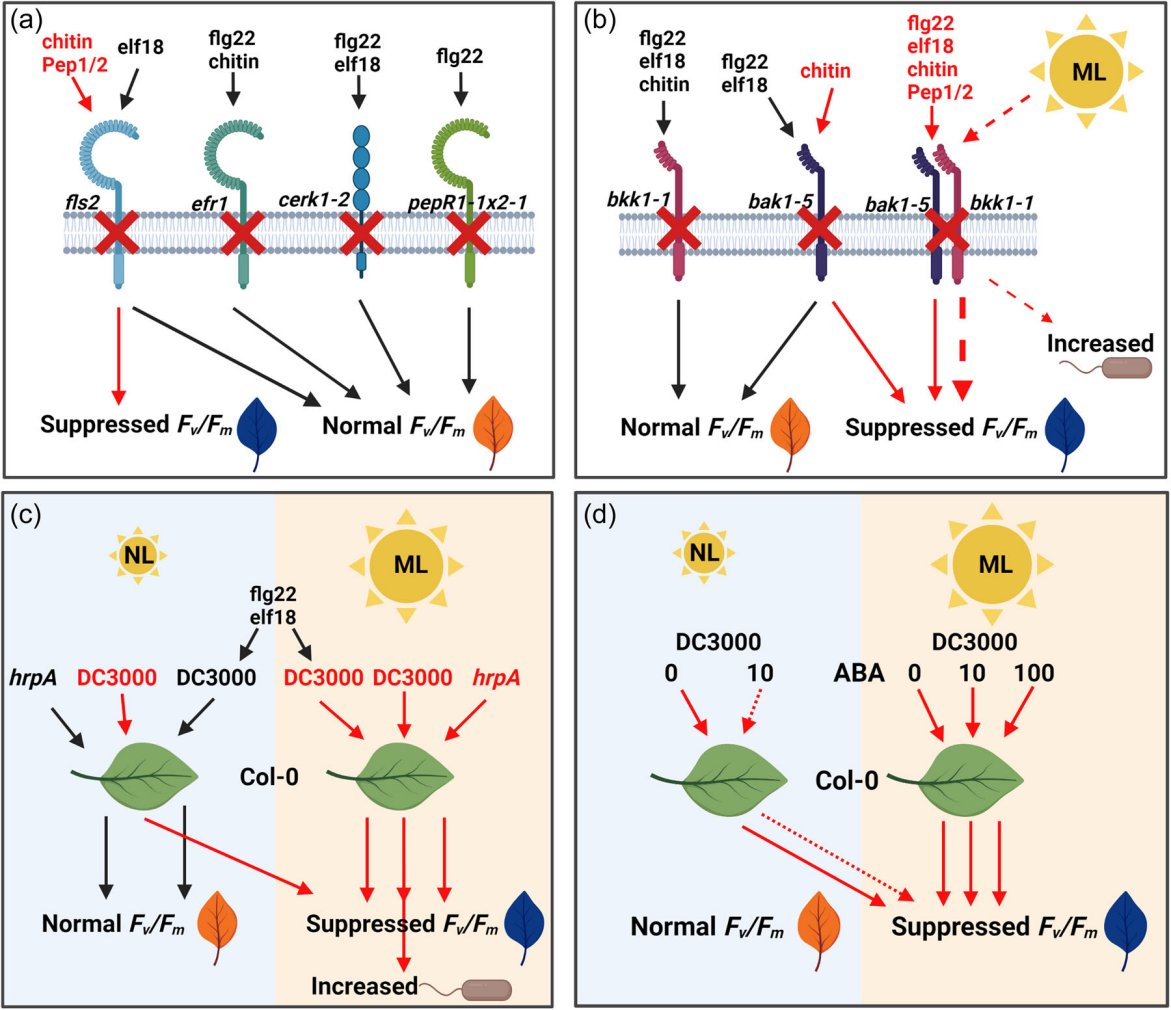
Schematic overview of findings from the study. Black arrows show pathways to normal ratio of variable/maximum fluorescence (*F*_v_/*F*_m_), while red arrows show pathways to suppressed *F*_v_/*F*_m_ or increased bacterial growth. (a) MAMP pretreatment followed by DC3000 infection on receptor mutant plants. *fls2* leaves pretreated with elf18 maintain normal *F*_v_/*F*_m_, while fls2 leaves pretreated with chitin, Pep1 or 2 show suppressed *F*_v_/*F*_m_. *efr1* leaves pretreated with flg22 or chitin maintain normal *F*_v_/*F*_m_. *cerk1‐2* leaves pretreated with flg22 or elf18 maintain normal *F*_v_/*F*_m_. *PepR1‐1 × 2‐1* leaves with flg22 maintain normal *F*_v_/*F*_m_. (b) MAMP pretreatment followed by DC3000 infection on MTI coreceptor mutant plants. *bak1‐5* leaves pretreated with flg22 or elf18 maintain normal *F*_v_/*F*_m_, while *bak1‐5* leaves pretreated with chitin show suppressed *F*_v_/*F*_m_. *bkk1‐1* leaves pretreated with flg22, elf18 or chitin maintain normal *F*_v_/*F*_m_. *bak1‐5/bkk1‐1* leaves pretreated with flg22, elf18, chitin, Pep1 or 2 show suppressed *F*_v_/*F*_m_. Under moderate light (ML) conditions (dashed red line), *bak1‐5/bkk1‐1* leaves show an increased suppression of *F*_v_/*F*_m_ (thick, red dashed arrow) and increased bacterial growth compared to normal light (NL) conditions. (c) Chlorophyll fluorescence and bacterial growth are altered under different light conditions. Under normal light (NL; 120 µmol m^−2^ s^−1^) conditions DC3000*hrpA*‐infected leaves maintain normal *F*_v_/*F*_m_, while DC3000‐infected leaves show suppressed *F*_v_/*F*_m_. Pretreatment of Col‐0 leaves with flg22 or elf18 under NL results in normal *F*_v_/*F*_m_. Under moderate light (ML; 650 µmol m^−2^ s^−1^) DC3000*hrpA*, DC3000 and flg22 or elf18 pretreated DC3000‐infected leaves all show suppressed *F*_v_/*F*_m_ and DC3000‐infected leaves show an increase in bacterial growth. (d) Chlorophyll fluorescence is reduced during moderate light and ABA treatment. Under normal light (NL; 120 µmol m^−2^ s^−1^) conditions DC3000‐infected leaves and leaves coinfiltrated with DC3000+ 10 µM show suppressed *F*_v_/*F*_m_. Under moderate light (ML; 650 µmol m^−2^ s^−1^) leaves infected with DC3000, leaves coinfiltrated with DC3000+ 10 µM and leaves coinfiltrated with DC3000+ 100 µM all showed suppressed *F*_v_/*F*_m_. Created with BioRender.com. [Color figure can be viewed at wileyonlinelibrary.com]

Priming of plants to reduce bacterial colonization has been previously demonstrated. Zipfel et al. (2006) showed that *A. thaliana* Col‐0 plants primed with flg22 or elf18 have reduced bacterial growth after infection with DC3000 compared to mock primed plants. In addition, Wan et al. (2008) showed that chitin pretreatment also protects *A. thaliana* against DC3000 multiplication. Thus, PRRs signal via a common pathway to induce MTI responses such as callose deposition, ROS and MAP Kinase activation. Activated MTI functions across pathogen classes, for example, the fungal MAMP chitin can prime a plant against bacterial infection (Nühse et al., 2000). Here we investigated the impact of such priming on the chloroplast as photosynthetic genes are significantly altered during disease and early immune signalling (Kachroo et al., 2021; Littlejohn et al., 2021). Our data show that priming with flg22, elf18 or chitin fully attenuates suppression of the maximum dark‐adapted quantum efﬁciency of PSII (*F*_v_/*F*_m_) by DC3000. Experiments with the broad‐spectrum ROS and NOS (reactive nitrogen species) stain H_2_DCF‐DA show that this protection extends in part to restricting DC3000 suppression of cROS within primed leaves (Figure 3) (de Torres Zabala et al., 2015). In general, plants that have lost a MAMP; FLS2, EFR, CERK1‐2 or DAMP receptor, PepR1‐1 × 2‐1, can sustain normal *F*_v_/*F*_m_ during bacterial infection by priming with an alternative M/DAMP that is, *efr*, *cerk1‐2* and *pepR1‐1 × 2‐1* plants retain normal *F*_v_/*F*_m_ with flg22 pretreatment (Figures 1, 2, 5e,f and 9). We did observe, however, that Pep1 and 3 provided reduced attenuation of *F*_v_/*F*_m_ suppression in *fls2* plants compared to Col‐0 (Supporting Information: Figure 2), and unexpectedly, chitin failed to protect *F*_v_/*F*_m_ suppression in *fls2* plants (Figures 2e,f and 9), whereas *efr* plants are protected by chitin treatment (Figure 2c,d and 9). These findings highlight specificity between initial downstream signalling through different PRRs. Our data suggest that the activation of immune signals transduced by PepR1/PepR2 and CERK1 are possibly not sufficiently strong to protect against bacterial infection in the absence of FLS2. Notably, in *efr* mutants pretreated with chitin, FLS2 activation could over‐ride those chloroplast processes targeted by bacteria during infection, indicating that there may be a requirement for preformed complexes with coreceptors to attenuate chloroplast immune priming.

PRRs represent the first line of induced defence and most require homo or heterodimerisation with a receptor for effective immune signalling. Chitin induces the dimerization and crosslinking of AtCERK1 which is required for immune signalling (Liu et al., 2012). By comparison, FLS2 and EFR are known to interact with co‐receptors BAK1 or BKK1, members of the SERK (SOMATIC EMBRYOGENESIS RECEPTOR KINASEs) protein family. Perception of flg22 or elf18 by their ligand leads to phosphorylation of their intracellular kinase domains and induction of downstream immune signals (Zhang & Zhou, 2010). BAK1 was originally identified as the coreceptor for the BR cell surface receptor BRI1. MTI is impaired in *bak1‐5* in response to flg22, elf18 or Pep1, leading to a reduced ROS burst and dampened MAPK activation (Roux et al., 2011). However, this mutant is not impaired in BR signalling (Schwessinger et al., 2011). In contrast, the ROS burst and MAPK responses to flg22, elf18 or Pep1 elicitation in a loss of function *bkk1‐1* mutant are similar to wild type (Roux et al., 2011). Priming of either *bkk1‐1* or *bak1‐5* individual mutants with flg22 and elf18 shows no suppression of *F*_v_/*F*_m_, indicating that these peptides can protect the PSII function from bacterial infection (Figures 4 and 9). This is comparable to the immune response functions observed for *bkk1‐1* and the BR responses observed for *bak1‐5* (Roux et al., 2011; Schwessinger et al., 2011). Chitin peptide priming prevented *F*_v_/*F*_m_ suppression by DC3000 in *bkk1‐1* plants but provided only partial protection in *bak1‐5* plants (Supporting Information: Figure 1a–d and Figure 9), consistent with the compromised immune signalling in *bak1‐5*. The double mutant *bkk1‐1/bak1‐5* has dramatically reduced immune responses to flg22 and elf18 elicitation (Roux et al., 2011). Here we demonstrated that at the level of chloroplast function, priming with flg22, elf18 or chitin offered no protection. Rather, we measure a quantitative hyper‐reduction in *F*_v_/*F*_m_ in comparison to Col‐0 plants (Figures 4 and 9 and Supporting Information: Figure 1d,e). The fact that chitin only provided partial protection to *bak1‐5* and no protection to *bkk1‐1/bak1‐5* plants is of interest since, to date, the LysM containing chitin receptor CERK1 is not known to use BAK1 or BKK1 for signalling (Liu et al., 2012; Yasuda et al., 2017). These data suggest that additional downstream signals linked to BAK1 are required for CERK1 signalling.

Both the chloroplast and light have an impact on plant resistance. Plants were grown under a low Red:Far‐Red ratio, which plants use to detect the proximity of neighbours, are more susceptible to pathogen infection by insects, biotrophic bacteria and necrotrophic fungi (Cerrudo et al., 2012; De Wit et al., 2013; Izaguirre et al., 2006; Moreno et al., 2009) due to the alteration of the defence hormones JA and SA. Exposure of plants to different light wavelengths has also been explored in plant defence. Tomato plants that were exposed to green and red light were more resistant to *Pseudomonas cichorii* JBC1 due to the up‐regulation of defence‐related genes (Nagendran & Lee, 2015). In addition, nightly red light treatment of tomato plants increased resistance to *P. syringae* DC3000 infection linked to the increased accumulation of SA (Yang et al., 2015). Exposure of plants to increasing light intensities causes rapid changes in nuclear gene expression in a photosynthesis‐dependent manner and is associated with chloroplast‐to‐nucleus retrograde signalling (Exposito‐Rodriguez et al., 2017; Suzuki et al., 2012; Vogel et al., 2014). A 1 h high light treatment of *Nicotiana benthamiana* reduced *F*_v_/*F*_m_ from 0.7 to 0.5 (Exposito‐Rodriguez et al., 2017), a much more significant drop than we see with *A. thaliana* over a 3.5 h period (Figure 6c), most likely associated with the higher light intensity of 1000 µmol m^−2^ s^−1^ compared to 650 µmol m^−2^ s^−1^ used in this study. Notably, this drop in *F*_v_/*F*_m_ was accompanied by a 50% increase in H_2_O_2_ (Exposito‐Rodriguez et al., 2017). A genetically encoded H_2_O_2_ reporter localised to the stroma and nucleus revealed that high light treatment (1000 µmol m^−2^ s^−1^) induced H_2_O_2_ production in these organelles for up to 1 h, and critically, the increase in nuclear H_2_O_2_ was dependent on electrons from the chloroplast (Exposito‐Rodriguez et al., 2017). High light conditions also induced perinuclear clustering of seven to eight chloroplast per nucleus, a similar observation as has been reported for plant–virus interactions (Caplan et al., 2015; Ding et al., 2019). It is predicted that this physical localization facilitates the rapid diffusion of H_2_O_2_ from chloroplast to nucleus, which elicits an alteration in nuclear gene expression (Exposito‐Rodriguez et al., 2017), and it would be interesting to compare the differences in these parameters between DC3000 challenged leaves at 120 and 650 µmol m^−2^ s^−1^.

While excess light is classically associated with enhanced resistance (Kangasjarvi et al., 2012; Karpinski et al., 2013) under moderate light, we unexpectedly found a synergistic effect with effector‐mediated suppression of *F*_v_/*F*_m_. Critically, MAMP pretreatment or ABA coinfiltration fails to attenuate this suppression during DC3000 infection (Figures 6a–f, 7f,g and 9). *F*_v_/*F*_m_ levels during a DC3000*hrpA* infection also reduced significantly during the first 6 h of moderate light but recovered to 0.7, compared to 0.75 under normal light (Figure 6a–d). Furthermore, the coreceptor double mutant, *bkk1‐1/bak1‐5*, also showed increased *F*_v_/*F*_m_ suppression compared to wild type following DC3000 challenge, which was accentuated under moderate light (Figure 6g,h). Strikingly, contrary to expectations given the elevated H_2_O_2_ production, Col‐0, *aao3* and *bkk1‐1/bak1‐5* lines all showed a significant increase in bacterial growth under moderate light but the hypersensitive *abi1/abi2/hab1* mutant and flg22‐insensitive mutant *fls2* showed no increase in susceptibility (Figures 7 and 8c). How and why these lines are insensitive to these elevated light conditions warrant further investigation.

Complex plant hormone synthesis and signalling crosstalk play an important role in the outcome of plant disease and defence responses. Both SA and JA are considered key hormones involved in plant immunity; however, it has become apparent in recent years that ABA has a significant role to play in hormone manipulation during pathogen infection (Robert‐Seilaniantz et al., 2011). Many organisms produce ABA, from cyanobacteria and fungi to humans, with kingdom‐specific synthesis pathways. In plants, ABA is synthesized from carotenoids within the chloroplast, with the final two enzymatic reactions in the cytosol (Finkelstein, 2013; Schwartz et al., 2003). As part of its virulence strategy, *P. syringae* induces de novo ABA biosynthesis in planta and this acts in part by suppressing SA biosynthesis and SA‐mediated defences to aid disease progression (Salomon et al., 2014; de Torres Zabala et al., 2007, 2009). Application of exogenous ABA (or coronatine) also induces the expression of the genes encoding three PP2Cs, HAI (HIGHLY ABA‐INDUCED) 1, HAI2 and HAI3, all of which interact with and inactivate MPK3 and MPK6, resulting in ABA‐mediated MPK3/MPK6 immune suppression (Mine et al., 2017). The PP2C triple mutant, *abi1/abi2/hab1*, is ABA hypersensitive and has enhanced susceptibility to DC3000, whereas the ABA biosynthetic mutant *aao3* shows enhanced disease resistance (de Torres Zabala et al., 2007, 2009). Chlorophyll fluorescence allows dissection of the dynamics of these mutants during DC3000 infection, with the triple mutant exhibiting a stronger suppression of *F*_v_/*F*_m_ (and a faster increase in NPQ), while the converse is true for the *aao3* mutant compared to Col‐0 (Figure 7a,b and Supporting Information: Figure 3) (de Torres Zabala et al., 2015). Notably, endogenous and exogenous ABA differentially impact apoplastic ROS production, with flg22 challenge of transgenic lines overexpressing ABA resulting in increased apoplastic H_2_O_2_ production, whereas plants with reduced ABA levels produced less apoplastic H_2_O_2_ following flg22 treatment (Tan et al., 2019). By contrast, ABA pretreatment resulted in a reduction in flg22‐induced apoplastic H_2_O_2_, indicating that endogenous and exogenous ABA function differently during MAMP‐induced apoplastic ROS burst in *A. thaliana* (Tan et al., 2019). During a DC3000*hrpA* infection, cROS is produced at 3–4 hpi, whereas DC3000 infection suppresses cROS, but not when primed with elf18 or flg22 (Figure 3a,b) (de Torres Zabala et al., 2015). Unexpectedly, cROS generation appeared ABA dose‐dependent, as leaves coinfiltrated with DC3000 and 10 µM ABA elicited a faster decrease of *F*_v_/*F*_m_ (similar to the hypersensitive *abi1/abi2/hab1* mutants).

Collectively, these data show that *F*_v_/*F*_m_ is a reliable, quantitative, real‐time indicator of pathogen infection and that abiotic factors affecting chloroplast functions, for example, light and ABA (induced during drought and other abiotic stresses) are generally associated with reduced tolerance to bacterial infection.

## CONFLICT OF INTEREST

The authors declare no conflict of interest.

## Supporting information

Supplementary information.

Supplementary information.

Supplementary information.

Supplementary information.

Supplementary information.
