# Supplementary figures and images for: Chloroplasts play a central role in facilitating MAMP‐triggered immunity, pathogen suppression of immunity and crosstalk with abiotic stress
Source: Plant Cell Environ. 2022 Aug 5;45(10):3001–17. doi: 10.1111/pce.14408 (PMC9544062; doi:10.1111/pce.14408)

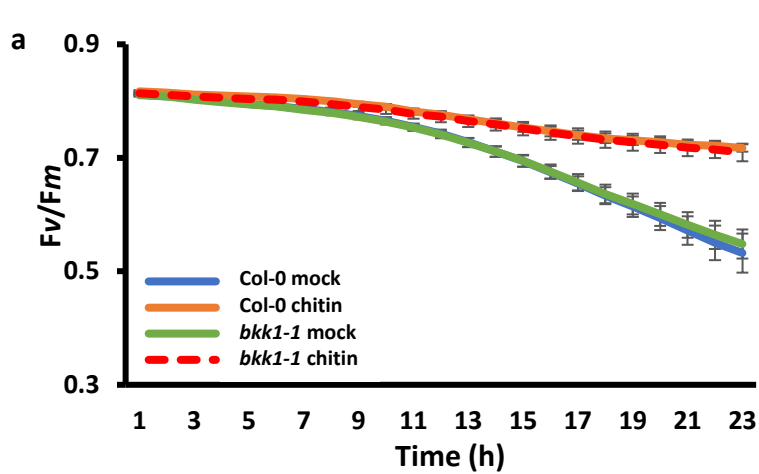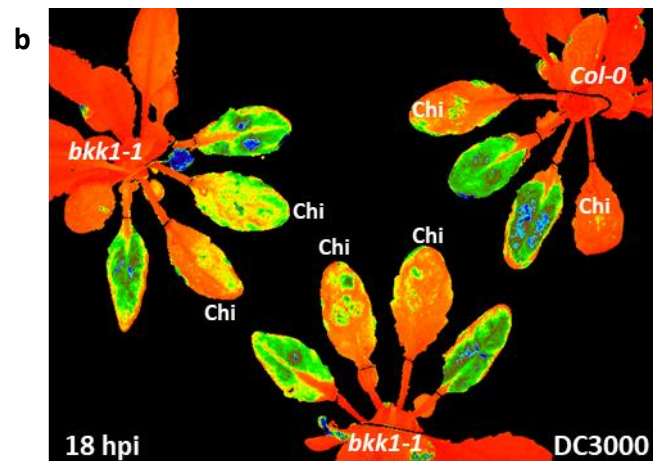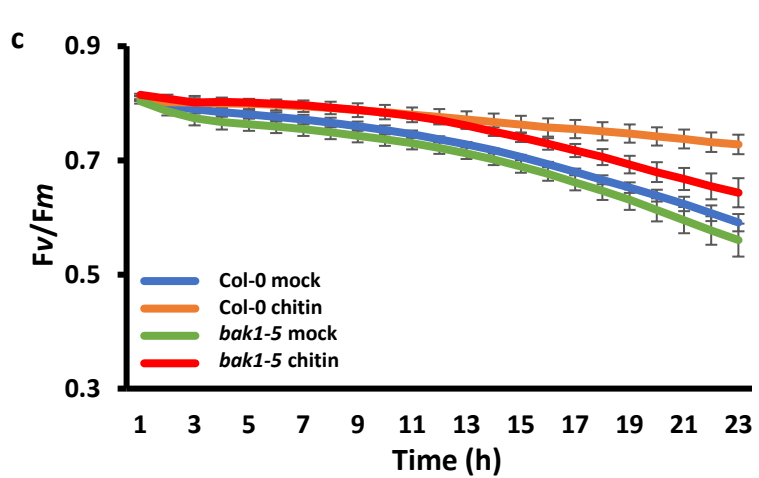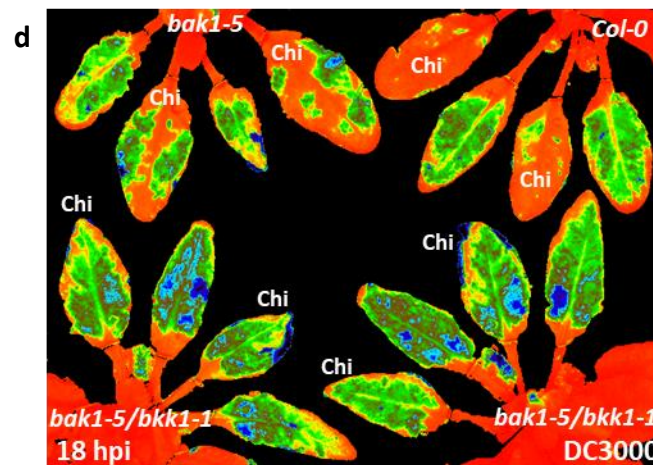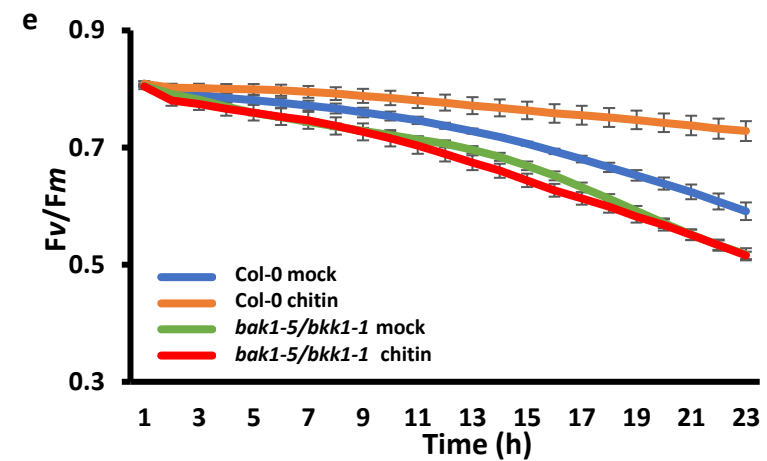

Supplement: Supplementary file 1 — Supplementary information. [file PCE-45-3001-s001.pdf]

a

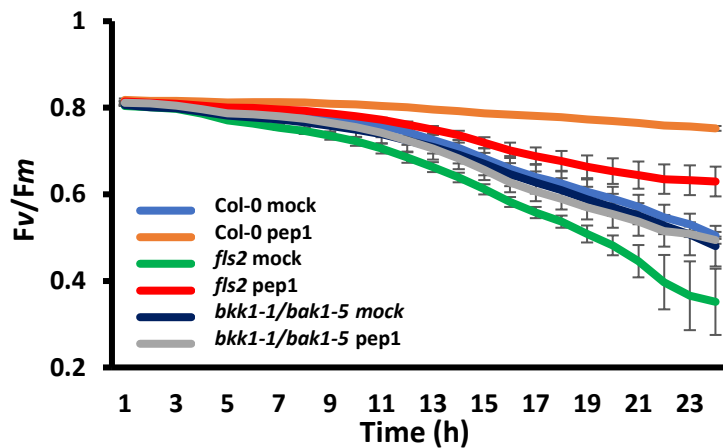

b

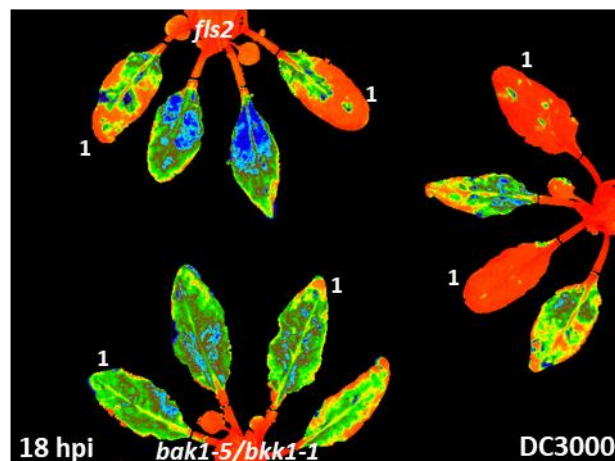

c

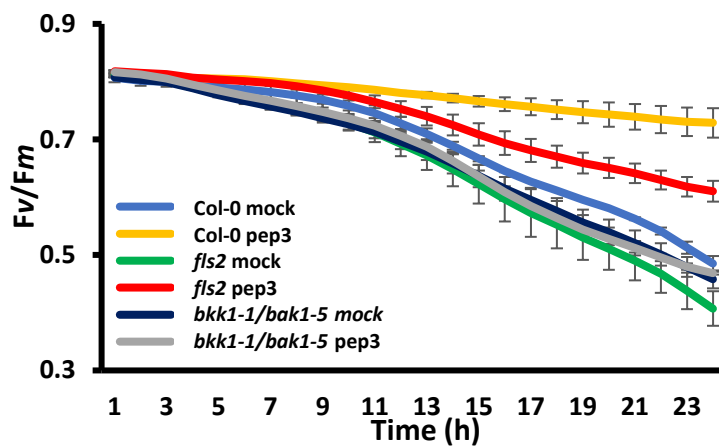

d

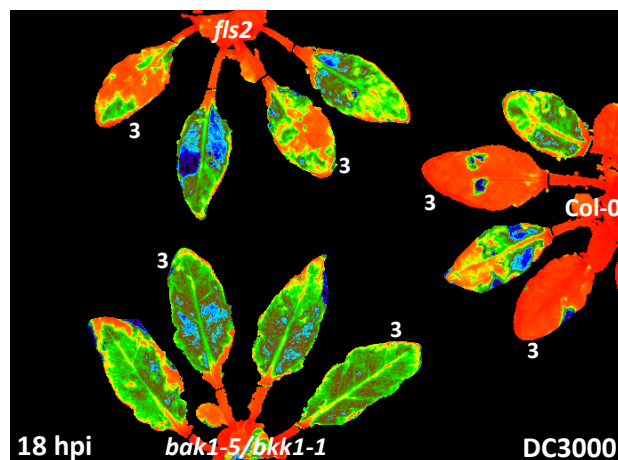

Supplement: Supplementary file 2 — Supplementary information. [file PCE-45-3001-s003.pdf]

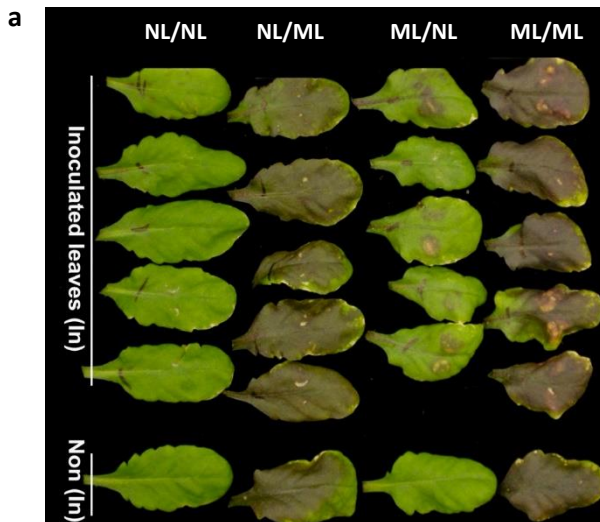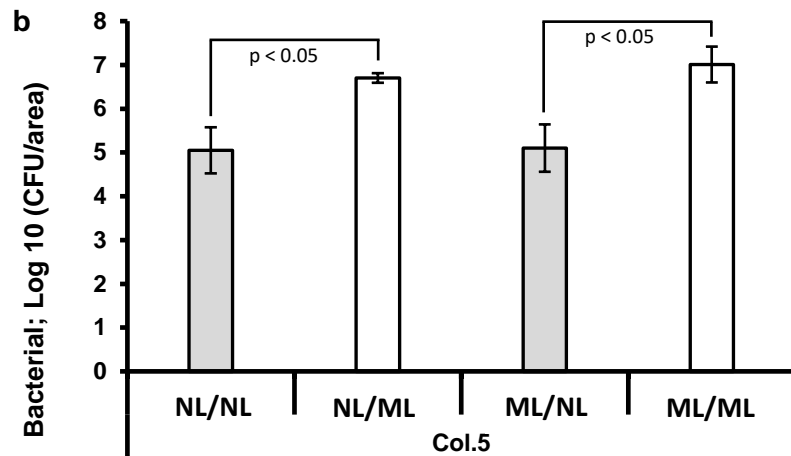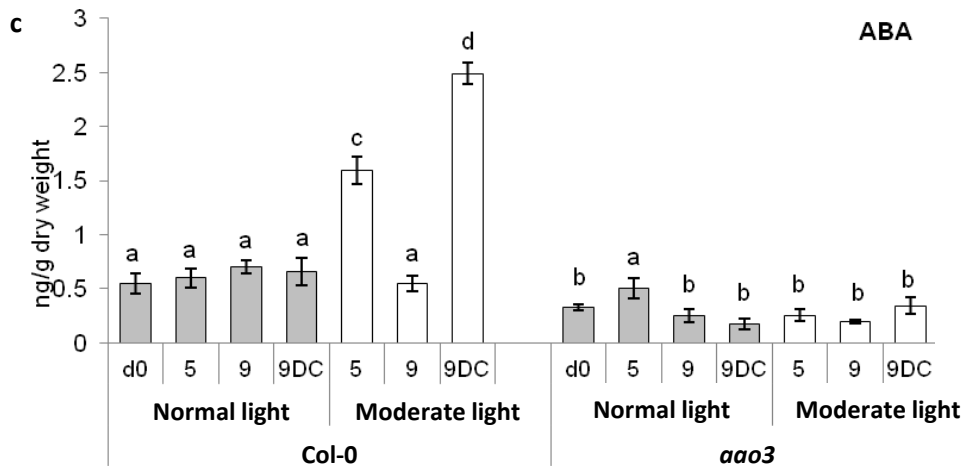

Supplement: Supplementary file 3 — Supplementary information. [file PCE-45-3001-s002.pdf]

a

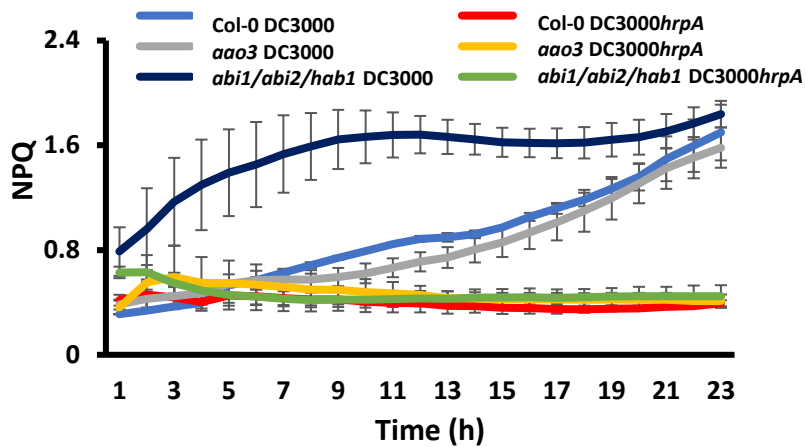

b

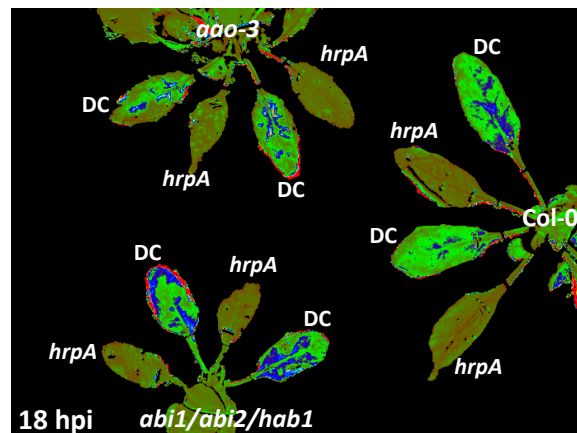

c

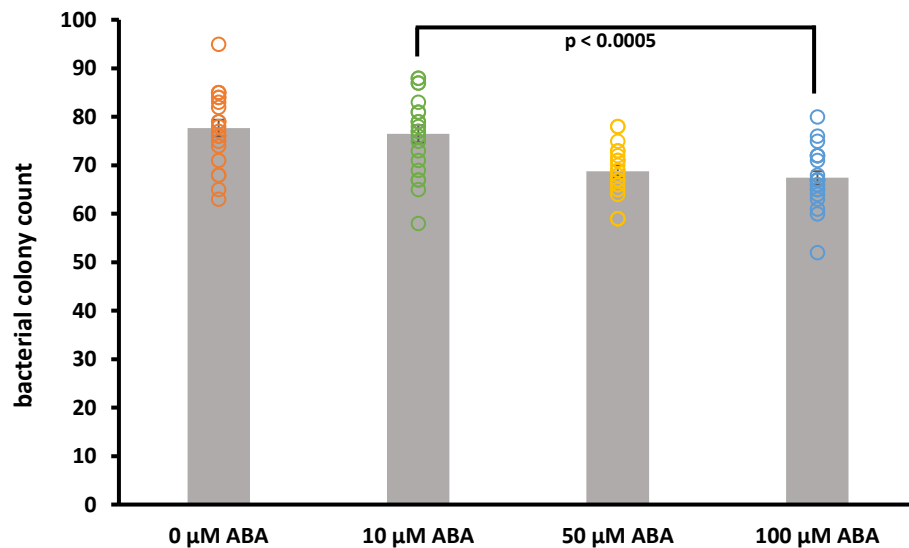

Supplement: Supplementary file 4 — Supplementary information. [file PCE-45-3001-s004.pdf]
