# Supplementary material for: Chloroplasts play a central role in facilitating MAMP‐triggered immunity, pathogen suppression of immunity and crosstalk with abiotic stress
Source: Plant Cell Environ. 2022 Aug 5;45(10):3001–17. doi: 10.1111/pce.14408 (PMC9544062; doi:10.1111/pce.14408)
Supplement: Supplementary file 5 — Supplementary information. [file PCE-45-3001-s005.docx]

**Supplementary Materials and Methods**

**Phytohormone measurements.** Three biological replicates were conducted containing two plants per replicate and three leaves per plant were harvested. Tissue was flash frozen in liquid nitrogen then freeze dried (Heto PowerDry LL3000). Dried samples were ground using a tissue lyser (Qiagen) then 10 mg of each sample was extracted in 400µl of 10% methanol 1% acetic acid containing internal standards; 2H4 SA (14.2 ng), 2H2 JA (10 ng), 2H6 ABA (2 ng) as previously described (de Torres Zabala *et al.*, 2015). Analyses was performed using an Agilent 6420B triple quadruple (QQQ) mass spectrometer (Technologies, Palo Alto, USA) as described (Page *et al.*, 2012). 10 µl of sample was loaded onto a Zorbax StableBond C18 1.8 mm, 2.1 x 100 mm reverse-phase analytical column (Agilent Technologies). Mobile phase A comprised 5% acetonitrile with 0.1% formic acid in water and mobile phase B was 95% acetonitrile with 0.1% formic acid in water. The data were extracted using MassHunter software (Agilent Technologies).

**Bacterial colony counts.** *Pseudomonas syringae* strains were grown overnight shaking (200 rpm) at 28°C in liquid Kings B containing appropriate antibiotics as described (Truman, de Zabala and Grant, 2006). Cultures were serially diluted to an OD_600_ 0.00001 and spotted on Kings B media containing 0, 10, 50 and 100 µM ABA with appropriate antibiotics (n≥20). Cfu were counted after 24 h incubation at 28°C.

**Supplementary Figure Legends**

**Figure Supp 1: Chitin pre-treatment provides full and partial protection on *bkk1-1* and *bak1-5* single mutant lines respectively but fail to protect *bak1-5/bkk1-1* lines.** MAMP pre-treatment was infiltrated 16 h prior to bacterial challenge, bacteria were infiltrated into the leaves on the abaxial side using one infiltration site per side of leaf. **a.** Graph quantifying changes in *F_v_/F_m_* over 23 h of DC3000 infection on Col-0 and *bkk1-1* leaves. Blue represents Col-0 leaves pre-treated with H_2_O; orange - Col-0 leaves pre-treated with chitin (100 µg/ml); green - *bkk1-1* leaves pre-treated with H_2_O and the dashed red line shows *bkk1-1* leaves pre-treated with chitin (100 µg/ml) **b.** Image of *F_v_/F_m_* for Col-0 (top right) and *bkk1-1* plants pre-treated with H_2_O or chitin (100 µg/ml) 18 hpi with DC3000. Orange represents expected *F_v_/F_m_*, whereas yellow/green/blue represents suppressed *F_v_/F_m_*. **c.** Graph quantifying changes in *F_v_/F_m_* over 23 hpi following DC3000 challenge of Col-0 or *bak1-5* leaves. Blue represents Col-0 leaves pre-treated with H_2_O; orange - Col-0 leaves pre-treated with chitin (100 µg/ml); green - *bak1-5* leaves pre-treated with H_2_O and red corresponds to *bak1-5* leaves pre-treated with chitin (100 µg/ml) **d.** Image of *F_v_/F_m_* for Col-0 (top right), *bak1-5* (top left) and *bak1-5/bkk1-1* (bottom) plants pre-treated with H_2_O or chitin (100 µg/ml) 18 hpi with DC3000. **e.** Graph quantifying changes in *F_v_/F_m_* over 23 hpi with DC3000 on Col-0 and *bak1-5/bkk1-1* leaves. Blue represents Col-0 pre-treated with H_2_O; orange - Col-0 pre-treated with chitin (100 µg/ml); green - *bak1-5* pre-treated with H_2_O and red line shows *bak1-5* pre-treated with chitin (100 µg/ml).

**Figure Supp 2: DAMPs provide partial *F_v_/F_m_*** **protection in PTI mutants.** DAMP pre-treatment was infiltrated 16 h prior to bacterial challenge, bacteria were infiltrated into the leaves on the abaxial side using one infiltration site per side of leaf. **a.** Graph quantifying changes in *F_v_/F_m_* over 24 hpi with DC3000 on Col-0, *fls2* and *bak1-5/bkk1-1* leaves. Blue represents Col-0pre-treated with H_2_O; orange - Col-0 pre-treated with pep1 (1 µM); green - *fls2* pre-treated with H_2_O; red - *fls2* pre-treated with pep1 (1 µM); dark blue - *bak1-5/bkk1-1* pre-treated with H_2_O and grey corresponds to *bak1-5/bkk1-1* pre-treated with pep1 (1 µM). **b.** Image of *F_v_/F_m_* for Col-0 (right), *fls2* (top) and *bak1-5/bkk1-1* (bottom) plants pre-treated with H_2_O and pep1 (1 µM) 18 hpi with DC3000. Orange represents expected *F_v_/F_m_*, whereas yellow/green/blue represents suppressed *F_v_/F_m_*. **c.** Graph quantifying changes in *F_v_/F_m_* over 24 hpi following DC3000 infection of Col-0, *fls2* and *bak1-5/bkk1-1*. Blue represents Col-0 leaves pre-treated with H_2_O; orange - Col-0 leaves pre-treated with pep3 (1 µM); green - *fls2* leaves pre-treated with H_2_O; red - *fls2* leaves pre-treated with pep3 (1 µM); dark blue - *bak1-5/bkk1-1* leaves pre-treated with H_2_O and grey corresponds to *bak1-5/bkk1-1* leaves pre-treated with pep3 (1 µM). **b.** Image of *F_v_/F_m_* for a Col-0 plant (right), *fls2* (top) and *bak1-5/bkk1-1* (bottom) plants pre-treated with H_2_O and pep3 (1 µM) at 18 hpi with DC3000. Orange represents expected *F_v_/F_m_*, whereas yellow/green/blue represents suppressed *F_v_/F_m_*.

**Figure Supp 3: Moderate light treatment causes increased bacterial growth and ABA levels.** Col-5 plants subject to HL or NL for 5 days followed by 4 dpi of DC3000 low inoculum (OD_600_: 0.0002). **a.** Effect of light treatment on Col-5 leaves; 5d continuous normal light (NL; 120 µmol m^-2^s^-1^) or moderate light (ML; 600 µmol m^-2^s^-1^) period followed by 4 dpi of DC3000 infection at NL or ML, (NL/NL) or (ML/ML) respectively or 5d continuous ML or NL period followed by 4 dpi of DC3000 infection at the opposite light intensity, (NL/ML) or (ML/NL) respectively. **b.** Bacterial growth of DC3000 on Col-5 plants under 5d continuous normal light (NL; 120 µmol m^-2^s^-1^) or moderate light (ML; 600 µmol m^-2^s^-1^) period followed by 4 dpi of DC3000 infection at NL or ML, (NL/NL) or (ML/ML) respectively or 5d continuous ML or NL period followed by 4 dpi of DC3000 infection at the opposite light intensity, (NL/ML) or (ML/NL) respectively. Each treatment represents the mean of six biological replicates Error bars, mean ± standard deviation (n=6) student t-test determined statistical significance of P < 0.05. Three biological replicates were undertaken. **c.** ABA measured in DC3000 infected Col-0 and *aao3* under 120 µmol m^-2^s^-1^ or 600 µmol m^-2^s^-1^ . Samples were harvested at day 0, 5 d, 9 d and 9 dpi with DC3000 under both 120 µmol m^-2^s^-1^ and 600 µmol m^-2^s^-1^. ABA levels were determined by LC-MS. The bars represent the mean of three biological replicates comprising two plants per replicate. Labels “a, b, c, d” above the columns discriminate differences at a significance of P < 0.05 (Student’s t-test for pairwise comparison of non-treated and treated plants). Error bars represent one standard deviation.

**Figure Supp 4: Effector induced suppression of ABA enhances NPQ induction. a.** Graph quantifying changes in NPQ over 24 hpi following DC3000 and DC3000*hrpA* infection of Col-0, *aao3* or *abi1/abi2/hab1* leaves. Blue represents Col-0 infiltrated with DC3000; red - Col-0 infiltrated with DC3000*hrpA*; grey - *aao3* infiltrated with DC3000; yellow - *aao3* infiltrated with DC3000*hrpA*; dark blue - *abi1/abi2/hab1* infiltrated with DC3000 and green corresponds to *abi1/abi2/hab1* infiltrated with DC3000*hrpA*. **b.** NPQ image of Col-0, *aao3* and *abi1/abi2/hab1* plants 18 hpi with DC3000 or DC3000*hrpA*. Dark green leaves correspond to normal NPQ whereas light green/blue colouration illustrates leaves with induced NPQ. **c.** Bacterial growth of DC3000 on Kings B agar with increasing concentrations of ABA in the media. Grey bars plotted showing average growth with all datapoints plotted shown in, 0 µM; orange, 10 µM; green, 50 µM; yellow and 100 µM; blue. Error bars, mean ± SE (n=21), student t-test determined statistical significance of P < 0.0005 for 10 µM vs 100 µM. Representative of four biological replicates.
